# Large-scale patterns of self-reported mood and sleep across the day and week on an online cognitive-training platform

**DOI:** 10.1371/journal.pdig.0001754

**Published:** 2026-09-24

**Authors:** Reina A. Mendoza, Andrew S. Tubbs, Michael L. Perlis, Fabian-Xosé Fernandez, Michael A. Grandner

**Affiliations:** 1 Department of Psychology, University of Arizona College of Science, Tucson, Arizona, United States of America; 2 Department of Psychiatry, Washington University School of Medicine, St. Louis, Missouri, United States of America; 3 Department of Psychiatry, University of Pennsylvania, Philadelphia, Pennsylvania, United States of America; 4 Department of Psychiatry, University of Arizona College of Medicine, Tucson, Arizona, United States of America; Wollo University, ETHIOPIA

## Abstract

Mood follows a well-documented circadian rhythm. Sleep timing and duration are similarly shaped by biological and social factors. However, large-scale, real-world data on how these patterns vary across demographic groups remain limited. Prior studies often rely on laboratory data or social media linguistic analysis, which lack direct self-reports. This study examines hourly, weekly, and demographic trends in mood and sleep duration reports using one of the largest online datasets of directly self-reported behavioral health: 468,092 Lumosity users (aged 20–79) who reported daily mood (−2 to +2 scale) and sleep duration (≤5–9 + hours). We assessed variation by time of day, day of week, and interactions of age, gender, and race/ethnicity using ANOVA and linear regression. Mood was higher among users reporting in the afternoon and evening, and lower from late night through morning. Mood showed a U-shaped pattern with age, consistent throughout the week, and longer sleep duration was linked to better mood reports throughout the day and week. Sleep duration varied by time of report and by day of week, with longer durations in the afternoon/evening and on weekends. Women reported longer sleep overall, Black users reported shorter sleep, and sleep duration declined from young adulthood through midlife before rising slightly among older users. Higher mood was associated with longer sleep, and the weekday-weekend mood difference was larger among users reporting longer sleep. These findings demonstrate the value of large-scale digital data for public health insight, highlighting demographic disparities in sleep and the importance of midlife as a key intervention period. Because the sample is likely skewed toward higher educational attainment and health engagement, disparity estimates may understate true population-level differences. The late-night peak in negative affect aligns with literature on nocturnal suicide risk, though this nonclinical sample cannot directly speak to clinical risk or crisis-service need.

## Author summary

In this study, we used a very large real-world dataset from an online cognitive-training platform (Lumosity) to examine how people’s self-reported mood and sleep vary across the day and across the week. We found clear time-based patterns: mood was generally lower among people logging in late at night and in the morning, and higher among those logging in later in the day, while sleep duration also differed in predictable ways between weekdays and weekends, with longer sleep typically reported on weekends. We also found that mood and sleep were closely linked, with longer sleep generally associated with better mood. At the same time, these patterns were not the same for everyone: they varied by age, gender, and race/ethnicity, and some groups consistently reported shorter sleep, pointing to broader social influences on sleep health. We see this work as part of a broader effort to use large digital datasets to better understand everyday mental health and sleep in natural settings. These findings may help inform more targeted support for insomnia, mood-related difficulties, and overnight crisis care. Because our sample reflects users of a commercial app rather than the general population, these disparities may understate, rather than overstate, true differences across the broader population.

## Introduction

Mood exhibits a well-documented diurnal rhythm, and sleep timing and duration are similarly shaped by endogenous circadian mechanisms as well as social and behavioral factors. While mood fluctuates systematically across the day and night [1,2], sleep follows its own age- and context-dependent trajectories [3], with variability across individuals [4], demographics [5], and environmental conditions [6]. Investigating both mood and sleep rhythms at scale presents a valuable opportunity to understand how these patterns evolve across the lifespan and how they may influence overall well-being.

Mood states are characterized by their expression of positive versus negative affect, two independent dimensions of emotional experience [7–9]. Positive affect, associated with energy, motivation, and well-being, tends to peak in the middle of the day before declining into the evening [10–12]. By contrast, negative affect, linked to stress, irritability, and distress, follows an opposite pattern, increasing during the late-night hours (0:00–6:00) and diminishing after sunrise [13]. This asymmetry suggests that positive and negative affect are regulated by separate circadian processes, rather than existing as opposite poles of a single continuum [14–21].

Controlled in-lab studies have demonstrated that mood rhythms persist even in isolation from external time cues, providing strong evidence for endogenous regulation by the circadian system [1,11]. More recently, researchers have turned to social media datasets, particularly Twitter (rebranded X in 2023) and other language-based platforms, to study mood variation in naturalistic settings [12,22]. These large-scale studies confirm the midday peak in positive affect and the nighttime peak in negative affect, aligning with laboratory findings. However, social media datasets are inherently limited by their indirect nature; mood is inferred from text-based sentiment analysis rather than directly reported. Additionally, cultural norms, language structure, and individual differences introduce variability in how emotions are expressed online [23].

To overcome these limitations, direct self-reported mood assessments provide a more precise measure of daily affective variation. Lumosity, a widely used brain-training application, includes a mood assessment feature that prompts users to rate their current emotional state upon logging in. By collecting self-reported mood data from a large, naturalistic sample, Lumosity offers a unique opportunity to replicate and expand upon existing research on diurnal mood variation while also examining how mood covaries with sleep in everyday life. Sleep is especially important in this context because, like mood, it varies across the week and across the adult lifespan.

The association between aging and sleep is often described as a progressive decline in sleep duration, but this characterization obscures important distinctions among different dimensions of sleep. Objective studies using polysomnography and actigraphy consistently show age-related changes in sleep architecture and sleep continuity, including reductions in slow-wave sleep, increased wakefulness after sleep onset, greater sleep fragmentation, and earlier sleep timing [4,24]. These findings have contributed to the broader view that sleep deteriorates with age. However, large-scale epidemiological studies complicate this interpretation by showing that older adults, particularly those aged 65 years and older, do not necessarily report shorter sleep duration than younger adults and may report similar or even longer total sleep time [25,26].

This apparent contradiction is best understood as a difference in what each methodological tradition measures. Polysomnography and actigraphy are well suited to detecting physiological and temporal changes in sleep, including sleep-stage composition, sleep efficiency, awakenings, circadian timing, and fragmentation. Across the adult lifespan, these measures show that sleep becomes lighter and more fragmented with age, with reductions in slow-wave sleep and shifts toward earlier sleep timing [4,27–29]. By contrast, epidemiological surveys and app-based assessments typically capture self-reported sleep duration, which reflects perceived sleep amount and, indirectly, the opportunity to sleep, rather than sleep architecture *per se* [30–32]. An older adult may therefore report a similar or longer sleep duration than a younger adult while still experiencing more fragmented, lighter, or less efficient sleep.

Several social and behavioral factors may help explain this divergence. Later life is often accompanied by retirement, reduced work obligations, greater schedule flexibility, daytime napping, and altered expectations about sleep, any of which may collectively increase time available for rest even as physiological sleep continuity declines [33–36]. The relevant question for the present study is not whether aging preserves sleep architecture; objective evidence indicates that it does not. Rather, the question is whether self-reported sleep duration in everyday life follows the same monotonic decline that might be inferred from physiological studies of sleep aging. This distinction is essential for interpreting age-related sleep patterns in large naturalistic datasets. The Lumosity data are therefore best positioned to address age-related variation in perceived sleep duration and sleep opportunity across real-world contexts, not age-related preservation or deterioration of sleep architecture.

As with mood research, social media platforms have been increasingly used to study sleep patterns. Users often mention their sleep duration, sleep difficulties, or feelings of fatigue in social media posts, enabling researchers to infer sleep trends through computational text analysis [37]. Studies based on Twitter, for example, have provided insights into sleep deprivation patterns across different demographics [38–40], but this approach relies on self-initiated language use, which may skew toward individuals experiencing disrupted sleep rather than representing the general population. Additionally, linguistic analysis does not capture actual sleep timing or duration with high accuracy, limiting its application for validating epidemiological sleep trends.

On the other hand, online platforms that directly ask users to report their sleep duration, such as Lumosity’s self-reported sleep assessment, offer a seamless, naturalistic approach to sleep tracking. These data allow for large-scale, population-level analysis of sleep behaviors across a wide age spectrum, providing a unique opportunity to examine how sleep duration varies by age, gender, and other demographic factors.

Within these questions lies a convergence of temporal, behavioral, and demographic variability. Mood and sleep are not distributed randomly across the day or across the population; rather, they are shaped by circadian biology, social schedules, occupational demands, age, gender, and structural inequalities [41–44]. Laboratory studies provide strong evidence that affective state varies by circadian phase, but they are necessarily limited in scale and ecological breadth [1]. Conversely, large-scale online studies can capture behavior across naturalistic settings, but many rely on inferred emotional expression rather than direct self-report [10,45]. Epidemiological studies have documented demographic disparities in sleep duration and sleep health but often lack fine-grained temporal information about when reports are made [46]. The present study addresses this gap by examining directly reported mood and sleep duration across hour of day, day of week, and demographic groups in a large-scale digital dataset. In doing so, it allows established observations from sleep and public health research to be evaluated together in a single naturalistic context.

In this study, we analyzed self-reported data from Lumosity users through a cross-sectional design, examining time-of-day and weekly variation in mood and sleep duration, while also evaluating whether demographic differences previously described in laboratory and epidemiological studies are detectable in everyday digital behavior. Based on prior research, we hypothesized that self-reported mood would be higher among users logging in around midday and lower among those logging in during the late-night hours, a pattern that would be consistent with, though not a direct test of, established circadian rhythms of affect. We also explored whether mood and sleep duration varied by age. Because prior findings are mixed on age, we treated the direction of the age effect as an exploratory question. Moreover, we expected demographic factors, such as gender and race/ethnicity, to influence both mood expression and sleep duration, reflecting well-documented disparities. Importantly, because each user contributed a single report, this design captures differences among users who happened to log in at different times, rather than within-person change over time. Our results offer new insights into these topics, with potential implications for future research on public health and sleep-related disparities.

## Methods

### Ethics statement

Ethical and data-governance review was conducted through both Lumos Labs’ data-access procedures and the University of Arizona Institutional Review Board. Investigators submitted a formal application to Lumos Labs’ Human Cognition Project specifying the study hypotheses, requested variables, and analysis plan. Access was limited to the subset of Lumosity variables needed to address the research questions, including demographic variables and mood/sleep-related measures. The University of Arizona IRB reviewed the study submission and issued a formal “Not Human Research” determination for STUDY00001275 (“Lumosity”), as the investigators analyzed a limited, anonymized/de-identified dataset provided by Lumos Labs and did not interact with users or receive direct identifiers. For these reasons, individual informed consent was not obtained by the investigators and was not required. The research team did not attempt to re-identify users, and all results are reported in aggregate form.

### Participants

Deidentified Lumosity self-report data were provided by Lumos Labs, Inc. (San Francisco, CA) at our request and included records from 493,758 unique users who logged into the platform from 2013 to 2014. The dataset was compiled so that each user contributed only a single observation. Thus, the data do not represent repeated measurements, longitudinal follow-up, or multiple logins from the same individuals. Instead, each record corresponds to one unique Lumosity user sampled at one login time, with that user’s mood report, sleep-duration report, demographic characteristics, and time-of-report variables linked to the hour and weekday of that login. The dataset included self-reported mood and sleep duration, anonymized demographic variables including age, gender, and race/ethnicity, and time-related login variables including hour of day and weekday. Time of day was determined from the UTC timestamp stored in the Lumosity database at login. User log-in times were aligned with their respective time-zones, and these times were assigned to the hour in which they occurred (e.g., the 23:00 bin included all logins between 23:00 and 23:59). Race/ethnicity was provided as a single user-reported demographic field. Because the available response categories combined racial and ethnic labels rather than distinguishing race and ethnicity as separate variables, we refer to this field as race/ethnicity throughout the manuscript. These categories are interpreted as socially defined demographic descriptors and not as biological, genetic, or ancestry-based classifications.

Mood and sleep duration reports were collected as part of the user’s daily interaction with the brain-training platform and therefore were initiated by user login rather than by an externally scheduled ecological momentary assessment protocol. Mood ratings were prompted by the question “How do you feel today?” and rated along a 5-point scale ranging from –2 to +2 (“not great,” –2; “not good,” –1; neutral, 0; “good,” 1; “great,” 2), accompanied by a short series of emojis displaying frowns, neutral expressions, and smiles across the scale. This item was treated as a broad self-reported index of current affective valence rather than as a validated clinical measure of mood, depression, anxiety, positive affect, or negative affect. Sleep duration in the last sleep opportunity was assessed by the question “How many hours did you sleep last night?” and rated according to one of five bins: ≤ 5 hours, 6 hours, 7 hours, 8 hours, and 9 + hours. Because the lower and upper response options were unbounded, ≤ 5 and 9 + hour categories were coded as 5 and 9, respectively, for subsequent analysis. This measure was treated as a coarse estimate of self-reported sleep duration and does not provide information about sleep timing, sleep quality, sleep continuity, sleep architecture, insomnia symptoms, or objectively measured sleep.

The original dataset (S1 Table) was preprocessed to reduce model complexity and improve the stability of estimates when demographic variables were analyzed in combination with hour and weekday. Age was grouped into decade-based categories (e.g., 30–39 years) to capture broad developmental periods, such as young adulthood, midlife, and older adulthood. This approach led us to exclude users aged 15–19, because this interval did not align with the decade-based grouping scheme, as well as users aged 80–84 and 85–90 because of smaller sample sizes. Similarly, Native American and Pacific Islander categories were excluded from the race/ethnicity variable because sparse cell counts across temporal strata could produce unstable estimates and increase the risk of overinterpreting underpowered subgroup patterns. After these exclusions, the final analytic sample included 468,092 users (S1 Table).

We retained race/ethnicity as an exploratory social-demographic variable because racial and ethnic disparities in sleep duration and sleep health are well documented in population-based research. Such disparities are generally understood to reflect unequal exposure to social, occupational, environmental, and structural conditions that shape sleep opportunity, including work schedules, neighborhood conditions, discrimination, chronic stress, socioeconomic position, and access to healthcare [47]. The present dataset provides an opportunity to examine whether such disparities are observable in a large naturalistic digital sample and whether they vary across hour of day and day of week. Thus, these analyses were intended to describe temporal patterning in reported sleep duration and mood across socially defined demographic groups, not to imply intrinsic biological differences between groups.

### Analysis

**Fig 1:**
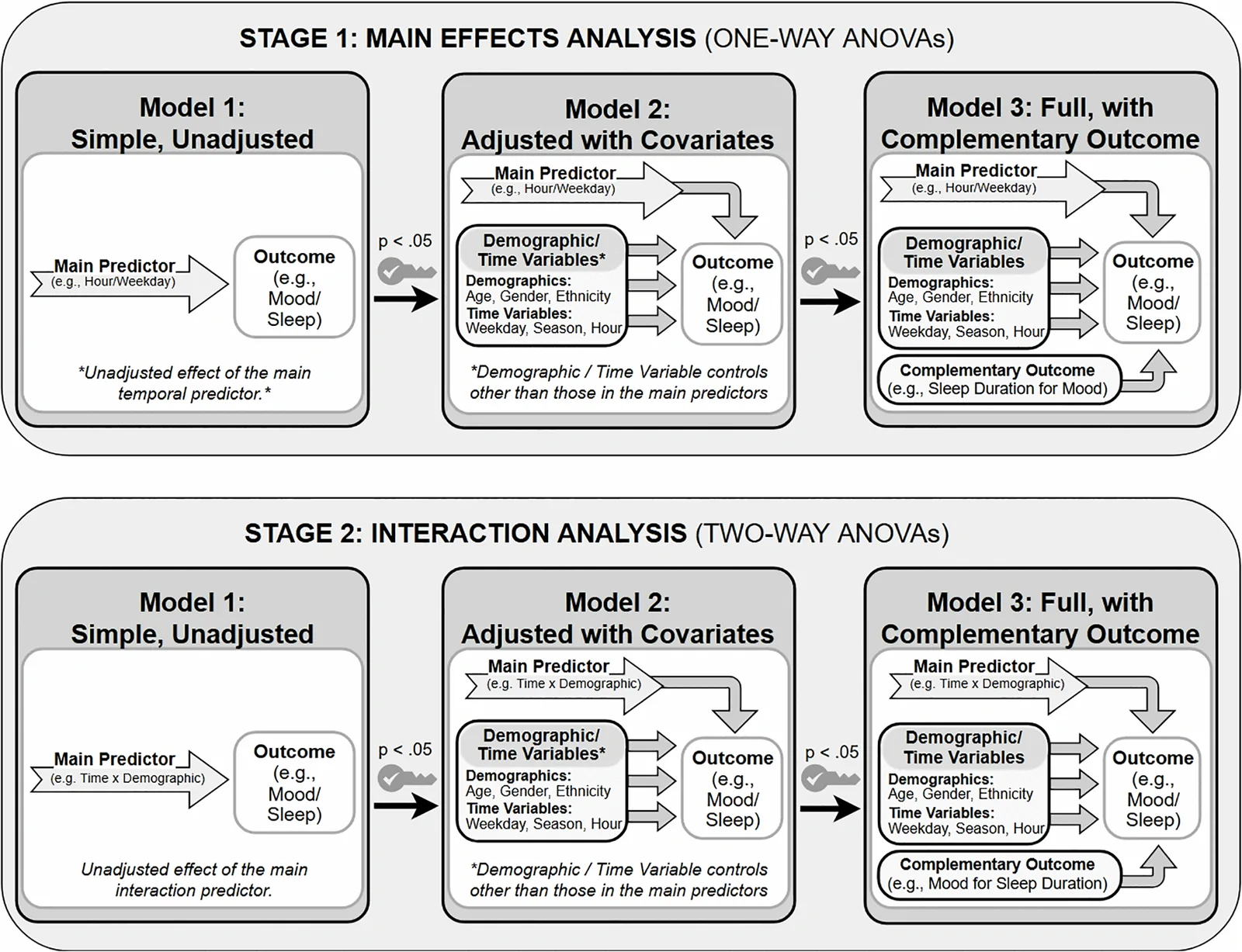
Three-step modeling approach for main effects and interactions.

To characterize hourly and weekly variation in mood and sleep duration reports, we used a three-step regression modeling strategy (Fig 1). Model 1 was an unadjusted model that included only the primary predictor of interest. Model 2 expanded this model by adding demographic and temporal covariates, including age, gender, race/ethnicity, weekday, and season. Model 3 further extended the adjusted model by including the complementary behavioral outcome as a categorical predictor: sleep duration in models predicting mood, and mood in models predicting sleep duration. For example, the fully specified model testing hour-of-day differences in mood included hour of day, demographic and temporal covariates, and categorical sleep duration. It is important to clarify the interpretive context of Model 3. Because mood and sleep duration are plausibly bidirectionally related, including one as a covariate when modeling the other does not represent a causally motivated adjustment, and we did not conduct a formal mediation or moderation analysis to establish the direction or mechanism of this relationship. Rather, Model 3 was included as a descriptive robustness check: to assess whether associations of interest (e.g., between hour of day or weekday and mood) persisted after accounting for the complementary behavioral outcome, given that both variables were measured concurrently and are known to covary. Coefficients from Model 3 should therefore be interpreted as evidence of robustness to this adjustment, not as causally identified effects of hour, weekday, or demographic variables net of mood or sleep duration. Across all models, mood and sleep duration were treated as interval outcomes, consistent with common practice in large-scale psychological research using symmetric response scales with approximately equal-interval interpretations [48,49].

Stage 1 analyzes the main effects of hour or weekday on mood and sleep duration with a set of three models. Subsequent models (1 → 2 → 3) are only performed if the previous one is significant. Model 1 is used for final coefficient extraction if all models are significant. Stage 2 analyzes the interaction terms of a time (hour/weekday) and demographic (age, gender, race/ethnicity) variable on mood and sleep duration. If a variable is already listed as a main predictor, it is not re-included in the controls of Models 2 and 3. Season was an adjusted variable, but not a predictor of interest.

Main effects of the primary temporal predictors, hour of day and weekday, were first evaluated using one-way ANOVAs. When a main effect was significant in the unadjusted model, we proceeded to the adjusted and fully specified models to determine whether the association remained robust after accounting for demographic, temporal, and complementary outcome variables. If the effect remained significant across all three models, coefficients were extracted from Model 1 to describe the unadjusted pattern of variation across the relevant hour or weekday categories.

The same sequential modeling strategy was used to evaluate interaction effects. For these analyses, interaction terms were created by pairing either hour of day or weekday with age, gender, race/ethnicity, or the complementary outcome in categorical form. Two-way ANOVAs were then used to test whether temporal variation in mood or sleep duration differed across these factors. As with the main-effect analyses, subsequent models were estimated only when the interaction remained significant in the preceding model. When an interaction was significant across all applicable models, coefficients and related statistics were extracted from Model 1 (similarly to the One-way tests) to characterize the unadjusted interaction pattern. Because these coefficients were extracted from a single prespecified model rather than from post-hoc pairwise comparisons among outcome groups, no additional correction for multiple comparisons was applied.

Type III sums of squares were used for all ANOVA tests to accommodate unequal group sizes across predictor categories. Because of the very large sample size, conventional assumption tests such as Levene’s test are likely to be overly sensitive to minor departures from homogeneity of variance. We therefore evaluated the robustness of the one-way ANOVA results by conducting corresponding Welch’s ANOVA tests, which are less dependent on the assumption of equal variances. Finally, we assessed multicollinearity among the predictors by fitting two regression models that included all variables used in the primary analyses: one model predicting mood and one model predicting sleep duration. Each model included age, gender, race/ethnicity, season, weekday, and the complementary outcome as a categorical predictor. Adjusted generalized variance inflation factor values were between 1.0 and 1.5 in both models, indicating no evidence of problematic multicollinearity. To complement the significance testing described above and address the limited practical interpretability of p-values with this sample size, we computed full (ω^2^) and partial omega-squared (ω^2^_p_) as a standardized effect size for each main effect and interaction term. Omega-squared was selected over eta-squared because eta-squared is known to be positively biased, particularly overestimating effect sizes, whereas omega-squared shows reduced bias and superior root-mean-square error performance relative to eta-squared [50]. Effect sizes were computed using the unadjusted (Model 1) version of each test, consistent with our approach of coefficient extraction described above. We recognize that the standard Cohen’s benchmarks for omega-squared (small ≈ .01, medium ≈ .06, large ≈ .14) are derived from sample sizes much smaller than those in the large-scale, naturalistic behavioral data presented here, and we therefore interpret them tentatively and relative to one another.

Weighted effect coding was applied to the hour and weekday variables to obtain a coefficient value for all terms relative to the weighted average, using two reference schemes across models (0:00 and 12:00 hours for hour-of-day models; Monday and Sunday for weekday models). We did not apply dummy coding, which requires selecting a single reference category and would frame each comparison as testing whether a given hour or weekday differs from that specific reference point. This would imply a directional hypothesis about particular clock times rather than a general hypothesis about variability across the day or week. By contrast, our hypothesis was that mood and sleep duration would vary within the day and across days, tested against a null hypothesis of no such variation. Weighted effect coding is more appropriate in this scenario because it compares individual clock hours and days to the grand average across hours and days.

All analyses were performed through R (version 4.4.3, R Foundation for Statistical Computing, Vienna, Austria) using the ‘wec’ and ‘car’ libraries, while preprocessing and data visualizations were created using Python (version 3.13) using the ‘pandas’, ‘numpy’, ‘scipy’, and ‘matplotlib’ packages.

## Results

Data from 468,092 users were analyzed for mood and sleep duration trends. Results begin by providing baseline hourly and weekly trends of mood or reported sleep duration, followed by interactions between these trends and other factors, including age, gender, race/ethnicity, and the complementary outcome (sleep to mood, vice versa). When “weighted average” is used, it refers to the average of the data across the time category (hour / weekday) from weighted effect coding. All one-way analyses involving hour of day and weekday were evaluated using corresponding Welch’s ANOVA tests to confirm that the results were robust to potential violations of homogeneity of variance.

### Hourly and weekly patterns of mood reports

**Fig 2:**
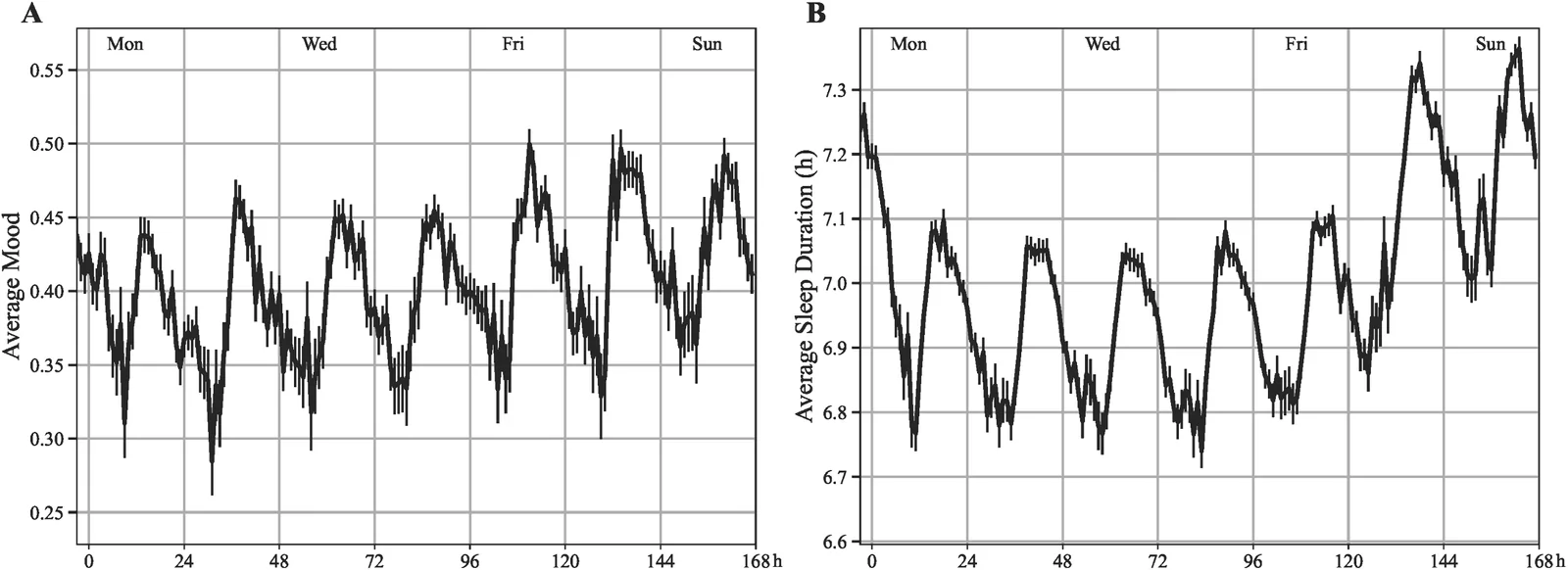
Time-of-day and weekly reports of average mood and sleep duration across all users. **(A**) Intraweek hourly mood ratings presented in sequential 168 clock-hour bins from Monday to Sunday (Mean ± SEM). Average mood ratings were lower early in the work week and higher among those reporting on Friday afternoons, with lower ratings again among users reporting from Sunday night to the following Monday. (**B**) Intraweek variation in sleep duration presented in sequential 168 clock-hour bins from Monday to Sunday (Mean ± SEM). Average reported sleep duration was longer among users reporting on Friday and Saturday nights and shorter earlier in the work week, with shorter reports overall on weekdays and longer reports on weekends.

Hour of day was a significant main effect in predicting mood reports (F(23, 468,068) = 41.83, p < .0001, *ω*^*2*^ = .002), and this association remained significant in both the adjusted and fully specified models (all p < .0001). In the regression model, coefficients were significant for all hour indicators except 11:00, 20:00, and 21:00. Relative to the weighted average mood level, estimates from 21:00–11:00 the following day were negative, whereas estimates from 12:00–20:00 were positive. Thus, hour of day was independently associated with mood (Fig 2), with higher-than-average mood reports occurring from afternoon into the evening and lower-than-average reports from the early night through the morning. We next examined whether this hourly pattern differed across demographic and behavioral factors.

To assess heterogeneity in these hourly mood patterns, two-way ANOVAs were used to examine interactions between hour of day and gender, race/ethnicity, age, and sleep duration in predicting mood reports (Table 1). A significant interaction was observed between gender and hour of day (F(23, 468,044) = 2.46, p < .001, *ω*^*2*^_p_ = 7.19 × 10^-5^) which remained significant in both the adjusted model (p < .001) and the fully specified model (p = .0065). Relative to male users, female users reported lower mood at 8:00 (*β* = −0.065, 95% CI [−0.100, −0.029], p < .001) and 9:00 (*β* = −0.063, 95% CI [−0.098, −0.027], p < .001). Mood reports were otherwise similar across the day, with female users showing modestly higher mood at 20:00 (*β* = 0.028, 95% CI [0.009, 0.046], p < .01) and 0:00 (*β* = 0.027, 95% CI [0.009, 0.046], p < .01). The effect size for the main effect of gender, however, was lower than the interaction’s (*ω*^*2*^_p_ = 2.84 × 10^-6^). An interaction between race/ethnicity and hour of day was significant in the unadjusted model (F(92, 467,972) = 1.27, p = .041, *ω*^*2*^_*p*_ = 5.31 × 10^-5^) but was no longer significant after adjustment (all p > .05).

**Table 1:** Model results for predicting mood reports in a 24 Hour / 7 day period.

	Model 1			Model 2		Model 3	
*Hour Models*	*F*	*P-value*	*ω*^*2*^ */ ω*^*2*^_*p*_	*F*	*P-value*	*F*	*P-value*
Hour	41.83	< .0001 ***	.002	35.91	< .0001 ***	19.41	< .0001 ***
Hour × Gender	2.46	< .001 **	7.19 × 10^-5^	2.39	< .001 **	1.88	.007 *
Hour × Race/Ethnicity	1.27	.041	5.31 × 10^-5^	1.24	.06	–	–
Hour × Age	1.21	.06	5.24 × 10^-5^	–	–	–	–
Hour × Sleep	1.93	< .0001 ***	.0002	1.86	< .0001 ***	–	–
*Weekday Models*	*F*	*P-value*	*ω*^*2*^ */ ω*^*2*^_*p*_	*F*	*P-value*	*F*	*P-value*
Weekday	34.27	< .0001 ***	.0004	30.25	< .0001 ***	14.99	< .0001 ***
Weekday × Gender	1.49	.18	6.28 × 10^-6^	–	–	–	–
Weekday × Race/Ethnicity	1.16	.263	8.41 × 10^-6^	–	–	–	–
Weekday × Age	1.55	.027 *	3.55 × 10^-5^	1.53	.03 *	1.11	.313
Weekday × Sleep	2.36	.0002 **	6.96 × 10^-5^	2.47	< .0001 ***	–	–

*Note: Each row represents a set of Type III F-tests for the respective main effect or interaction of interest. Model 1 is the unadjusted model, Model 2 includes demographic and time controls (gender, race/ethnicity, age, weekday, season), and Model 3 includes demographic controls, time controls, and sleep as a categorical control. Model 3 serves as a descriptive robustness check for whether associations persist when accounting for the complementary outcome and is not intended to represent a causally adjusted estimate. Subsequent models are only performed if the previous model is significant. Rows where sleep is included in the interaction do not have a Model 3 as sleep is already within the model.* * = < .05; ** = < .001; *** = < .0001.

**Fig 3:**
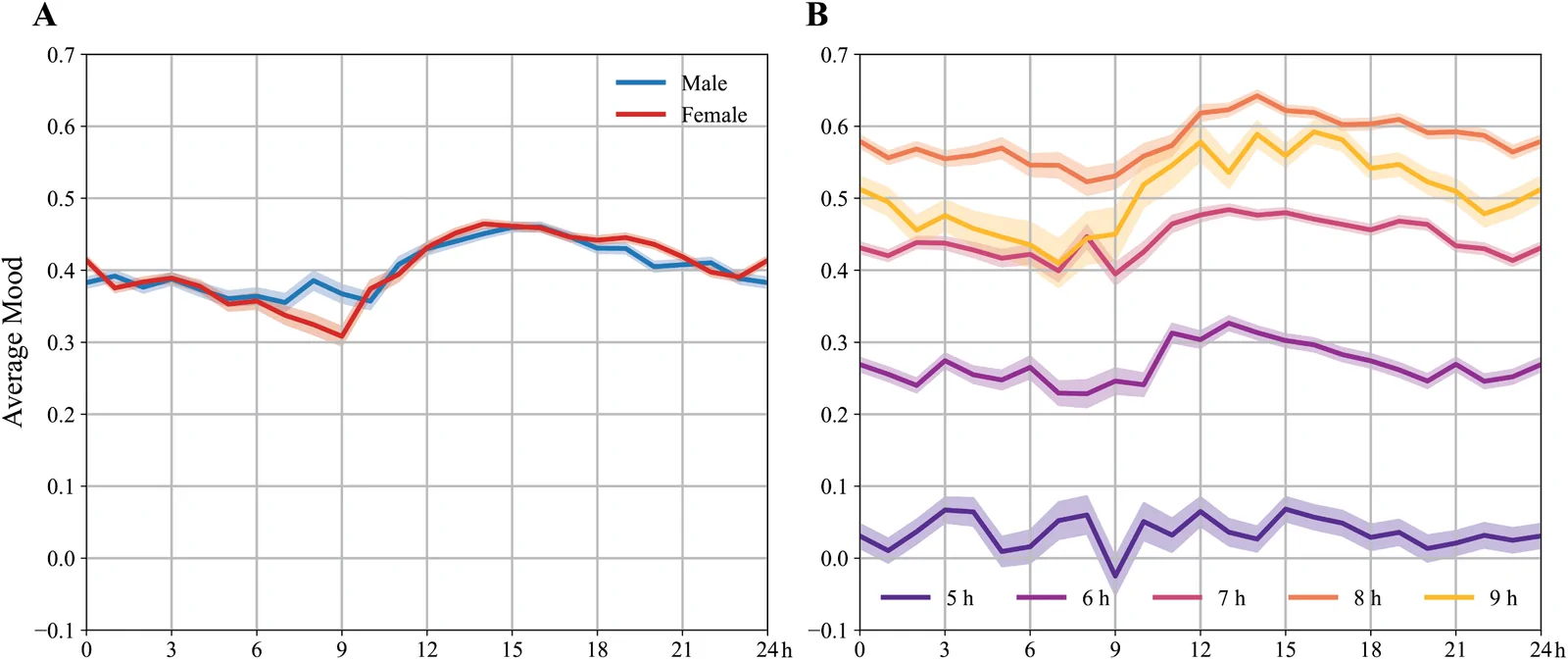
Gender and sleep duration variation in average hourly reports of mood. (**A**) Hourly mood ratings averaged across days, plotted for each hour bin and separated by gender. Mood ratings were similar for male and female users across most hours, with lower ratings among female users reporting in the morning and higher ratings among female users reporting in the late afternoon and at midnight. Both groups showed higher ratings among users reporting in the afternoon and lower ratings among those reporting overnight; among male users, ratings at 8:00 departed from this pattern relative to adjacent hours. (**B**) Hourly mood ratings averaged across days, plotted for each hour bin and separated by sleep duration category. Users reporting longer sleep duration reported higher overall mood, with the 8-hour group highest. Among users reporting 5 hours of sleep, hourly mood averages varied more sharply by report time; this variation was smaller among users reporting longer sleep durations. Users reporting 9 hours of sleep showed lower mood on average than the 8-hour group.

In contrast to gender, no significant interaction was observed between age and hour of day in the unadjusted model (F(115, 467,948) = 1.21, p = .06, *ω*^*2*^_*p*_ = 5.24 × 10^-5^). Age, however, was a significant main effect in predicting mood reports (F(95, 467,948) = 196.71, p < .0001); the omnibus effect size for age as a whole was *ω*^*2*^_*p*_ = .002, with mood declining across young and middle adulthood and increasing again in late adulthood. Sleep duration showed a robust interaction with hour of day in both the unadjusted model (F(92, 467,972) = 1.93, p < .0001, *ω*^*2*^_*p*_ = .0002) and the adjusted model (F(92, 467,953) = 1.86, p < .0001). Across the day, longer sleep duration was associated with higher reported mood, with 8 hours of sleep showing the largest positive difference relative to the 5-hour baseline (*β* = 0.553, 95% CI [0.545, 0.561], p < .0001). The main effect of sleep duration category on mood corresponded to *ω*^*2*^_*p*_ = .048, a substantially larger effect size than any of the temporal or demographic terms reported above. In addition, longer sleep duration was associated with more pronounced hourly variation in mood, whereas shorter sleep duration was associated with flatter and more irregular hourly patterns (Fig 3).

Beyond hourly variation, mood reports also exhibited systematic differences across the week. Weekday had a significant main effect in predicting mood reports (F(6, 468,085) = 34.27, p < .0001, *ω*^*2*^ = .0004), and this association remained significant in the adjusted (F(6, 468,049) = 30.25, p < .0001) and fully specified models (F(6, 468,045) = 14.99, p < .0001). In the regression model, coefficients for all weekdays were significant (all p < .05), with Monday through Thursday associated with lower-than-average mood and Friday through Sunday associated with higher-than-average mood relative to the weighted mean. Accordingly, mood reports increased toward the weekend and declined at the start of the subsequent week (Fig 2).

To determine whether these weekday effects varied across subgroups, two-way ANOVAs were conducted to examine interactions between weekday and gender, race/ethnicity, age, and sleep duration in predicting mood reports (Table 1). No significant interactions were observed between weekday and gender in the unadjusted model (F(6, 468,078) = 1.49, p = .18, *ω*^*2*^_*p*_ = 6.28 × 10^-6^), nor between weekday and race/ethnicity (F(24, 468,057) = 1.16, p = .263, *ω*^*2*^_*p*_ = 8.41 × 10^-6^). A significant interaction between age and weekday emerged in the unadjusted model (F(30, 468,050) = 1.55, p = .027, *ω*^*2*^_*p*_ = 3.55 × 10^-5^) and remained significant in the adjusted model (F(30, 468,019) = 1.53, p = .03); however, this interaction was no longer significant in the fully specified model (F(30, 468,015) = 1.11, p = .313).

**Fig 4:**
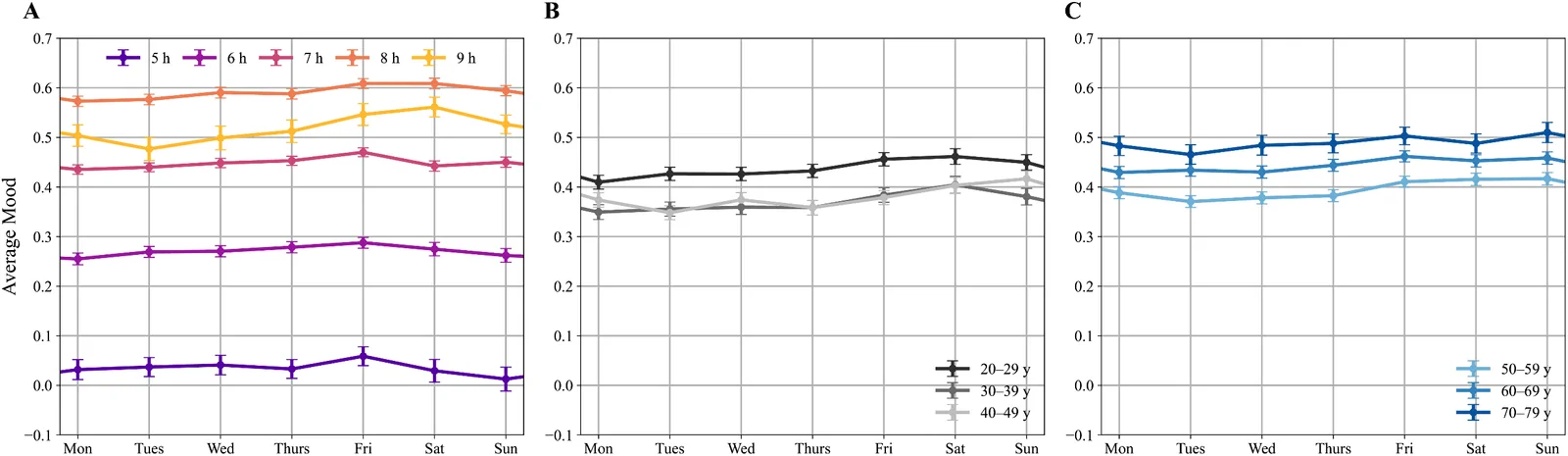
Age and sleep duration variation in average daily reports of mood. (**A**) Daily mood ratings averaged across all hours, plotted for each day and separated by sleep duration category. Users reporting longer sleep durations reported higher overall mood on average, with the 8-hour group highest. Among users in the 8-hour category, mood ratings were higher on Saturday and Sunday than on weekdays; this weekday-weekend difference was smaller or not evident in the other sleep-duration categories. Among users reporting 5 hours of sleep, weekend mood ratings were lower than in other sleep categories, approaching the neutral midpoint (0) of the mood scale. (**B–C**) Hourly variation in mood by age group (Mean ± SEM). Mood ratings showed distinct hourly patterns across age groups, with larger hour-to-hour differences among older adults (60–69 years; blue; 70–79 years, red) than among younger adults (20–29 years, grey). Across all age groups, ratings were higher among users reporting on midday hours (12:00–15:00) and lower among those reporting at night (21:00–6:00).

Consistent with earlier analyses, age alone exhibited a U-shaped association with mood reports (*ω^2^*_*p*_ = .002). Mood declined from early adulthood through midlife and then increased in later adulthood, returning to comparable levels at ages 60–69 (*β* = 0.007, 95% CI [0.000, 0.014], p < .05) and rising further at ages 70–79 (*β* = 0.050, 95% CI [0.042, 0.060], p < .0001; Fig 4). Extending this pattern, a significant interaction between sleep duration and weekday was observed in the unadjusted model (F(24, 468,057) = 2.36, p = .0002, *ω*^*2*^_*p*_ = 6.96 × 10^-5^) and remained significant in the adjusted model (F(24, 468,021) = 2.47, p < .0001). The omnibus main effect of sleep duration on mood predictions within this model was *ω*^*2*^_*p*_ = .049, similar to the effect size observed in the sleep duration and hour of day model. Consistent with prior models, longer sleep duration was associated with higher overall mood (all p < .0001), with peak mood observed at 8 hours of sleep. Furthermore, longer sleep duration was associated with larger weekday-weekend differences in mood, whereas shorter sleep duration was associated with minimal weekend variation (Fig 4).

### Hourly and weekly patterns of sleep duration reports

Hour of day, defined as the user’s login or report time, was significantly associated with reported sleep duration (F(23, 468,068) = 144.94, p < .0001, *ω*^*2*^ = .007), and this association remained significant in both the adjusted and fully specified models (all p < .0001). In the regression model, all hour indicators were significant except 0:00. Relative to the weighted average, users who logged in between 1:00 and 13:00 reported lower sleep duration, whereas users who logged in between 14:00 and 23:00 reported higher sleep duration. Thus, reported prior-night sleep duration varied systematically by time of report: users logging in from the middle of the night through late morning reported shorter sleep, while those logging in from the afternoon into the evening reported longer sleep (Fig 2). We next examined whether this time-of-report pattern differed across demographic and psychological factors.

**Fig 5:**
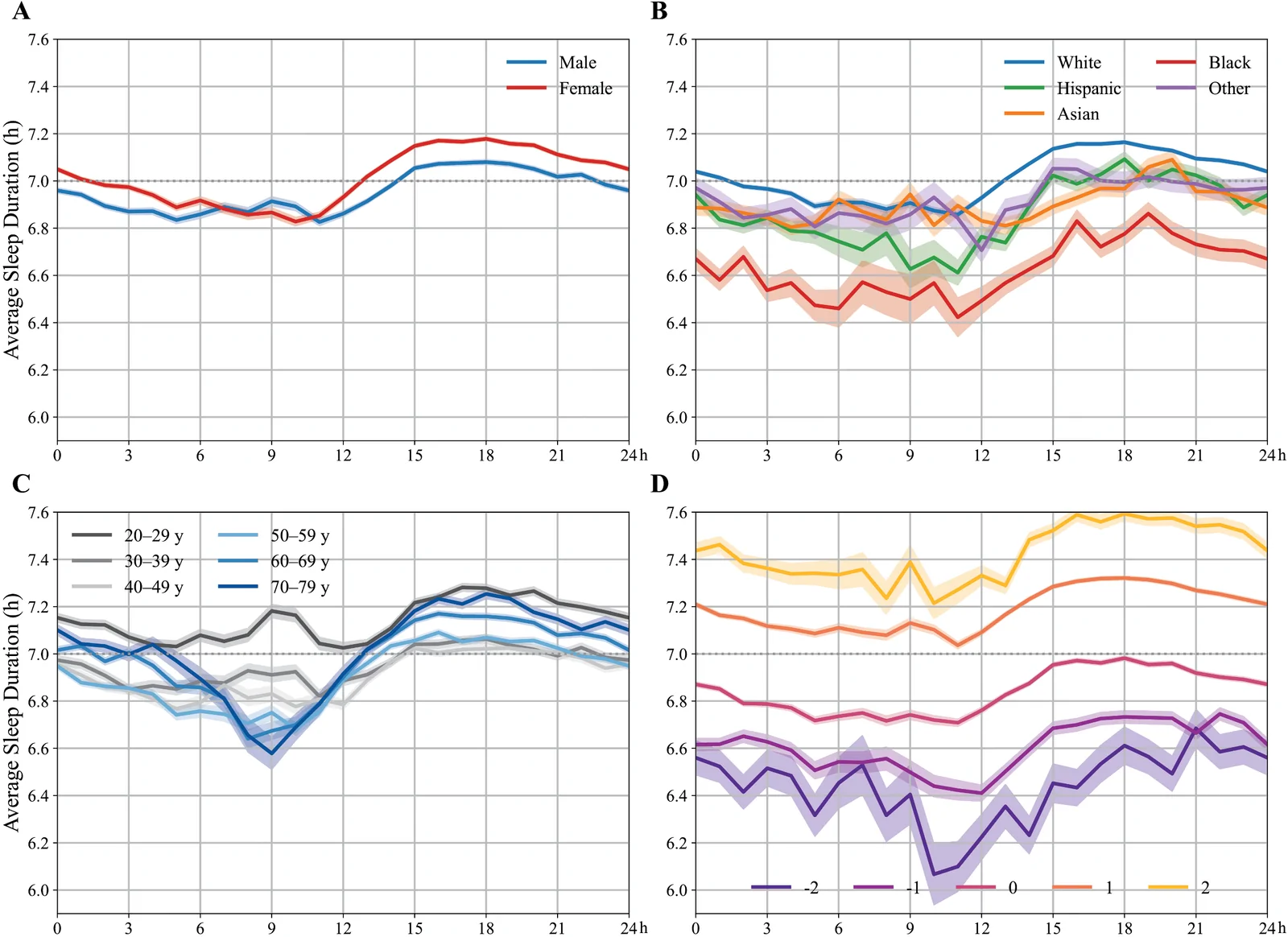
Gender, race/ethnicity, age, and sleep duration variation in average hourly reports of sleep duration. (A) Hourly sleep duration ratings averaged across days, plotted for each hour bin and separated by gender. Female users reported higher sleep durations on average, with a smaller difference during morning hours. Reported sleep durations were below 7 hours among users reporting during morning hours and above 7 hours among those reporting during afternoon hours. (B) Hourly sleep duration ratings averaged across days, plotted for each hour bin and separated by race/ethnicity. White users reported the highest sleep duration across the day; Hispanic, Asian, and Other users reported lower sleep durations, and Black users reported the lowest, with hourly averages that did not exceed 7 hours. (C) Hourly sleep duration ratings averaged across days, plotted for each hour bin and separated by age. Users aged 20–29 reported the highest sleep durations on average across most hours. Users aged 70–79 reported sleep durations similar to the 20–29 group in the afternoon, and the lowest sleep durations of any age group in the morning. (D) Hourly sleep duration ratings averaged across days, plotted for each hour bin and separated by mood category. Users reporting the highest mood rating (+2) reported the highest sleep durations across hours, with progressively lower sleep durations at lower mood ratings. Sleep duration reports were higher among users reporting during afternoon hours across mood categories. Users reporting the lowest mood rating (-2) reported the lowest sleep durations across all hours and the largest hour-to-hour differences among mood groups.

Two-way ANOVAs were conducted to assess interactions between hour of day and gender, race/ethnicity, age, and mood in predicting reported sleep duration (Table 2). A significant interaction was observed between gender and hour of day (F(23, 468,044) = 4.86, p < .0001, *ω*^*2*^_*p*_ = .0002), which remained significant in both the adjusted and fully specified models (all p < .0001; Fig 5). The omnibus main effect of gender had an effect size of *ω*^*2*^_*p*_ = .001. On average, female users reported longer sleep duration (*β* = 0.079, 95% CI [0.073, 0.086], p < .0001); however, they reported shorter sleep duration during morning hours, with the largest difference observed at 10:00 (*β* = −0.140, 95% CI [−0.192, −0.095], p < .0001).

**Table 2:** Model results for predicting sleep duration reports in a 24 hour / 7 day period.

	Model 1			Model 2		Model 3	
*Hour Models*	*F*	*P-value*	*ω*^*2*^ */ ω*^*2*^_*p*_	*F*	*P-value*	*F*	*P-value*
Hour	144.94	< .0001 ***	.007	129.12	< .0001 ***	115.54	< .0001 ***
Hour × Gender	4.86	< .0001 ***	.0002	4.25	< .0001 ***	3.78	< .0001 ***
Hour × Race/Ethnicity	2.25	< .0001 ***	.0002	2.22	< .0001 ***	2.11	< .0001 ***
Hour × Age	5.50	< .0001 ***	.001	5.38	< .0001 ***	5.25	< .0001 ***
Hour × Mood	2.02	< .0001 ***	.0002	1.98	< .0001 ***	–	–
*Weekday Models*	*F*	*P-value*	*ω*^*2*^ */ ω*^*2*^_*p*_	*F*	*P-value*	*F*	*P-value*
Weekday	590.85	< .0001 ***	.007	576.67	< .0001 ***	561.29	< .0001 ***
Weekday × Gender	7.36	< .0001 ***	8.15 × 10^-5^	6.98	< .0001 ***	7.40	< .0001 ***
Weekday × Race/Ethnicity	2.81	< .0001 ***	9.27 × 10^-5^	2.81	< .0001 ***	2.78	< .0001 ***
Weekday × Age	1.79	< .0001 ***	.001	1.89	< .0001 ***	18.80	< .0001 ***
Weekday × Mood	1.49	.059	2.50 × 10^-5^	–	–	–	–

*Each row represents a set of Type III F-tests for the respective main effect or interaction of interest. Model 1 is the unadjusted model, Model 2 includes demographic and time controls (gender, race/ethnicity, age, weekday, season), and Model 3 includes demographic controls, time controls, and mood as a categorical control. Model 3 serves as a descriptive robustness check for whether associations persist when accounting for the complementary outcome and is not intended to represent a causally adjusted estimate. Subsequent models are only performed if the previous model is significant. Rows where mood is included in the interaction do not have a Model 3 as mood is already within the model.* * = < .05; ** = < .001; *** = < .0001.

In addition to gender, a significant interaction between race/ethnicity and hour of day was observed (F(92, 467,972) = 2.25, p < .0001, *ω*^*2*^_*p*_ = .0002) and remained significant in both the adjusted and fully specified models (all p < .0001). The omnibus main effect of race/ethnicity was *ω*^*2*^_*p*_ = .004. Relative to White users, reported sleep duration was lower among Black users (*β* = −0.390, 95% CI [−0.407, −0.366], p < .0001), followed by Hispanic users (*β* = −0.140, 95% CI [−0.159, −0.130], p < .0001), Asian users (*β* = −0.140, 95% CI [−0.153, −0.124], p < .0001), and users in the Other category (*β* = −0.110, 95% CI [−0.131, −0.094], p < .0001). Hourly sleep-duration profiles differed across groups, with White users exhibiting more consistent time-of-report patterns and Black users showing greater amplitude of hourly variation (Fig 5).

Age also moderated the association between hour-of-day and reported sleep duration, with a significant interaction observed in predicting sleep duration (F(115, 467,948) = 5.50, p < .0001, *ω*^*2*^_*p*_ = .001), and this interaction remained significant in both the adjusted and fully specified models (all p < .0001). The omnibus main effect for age had an effect size of *ω*^*2*^_*p*_ = .005. Across age groups, sleep duration followed a U-shaped pattern, with users aged 20–29 reporting the highest overall sleep duration, followed by declines across successive decades and increases again among users aged 60–79 (all p < .0001). Notably, greater between-group differences were evident during morning hours: users aged 20–29 reported higher sleep duration in the morning compared with most other age groups, with the exception of users aged 70–79 (Fig 5). Nevertheless, this time-of-report pattern in sleep duration was present across all age groups.

Beyond demographic characteristics, a significant interaction was observed between mood and hour of day (F(92, 467,972) = 2.02, p < .0001, *ω*^*2*^_*p*_ = .0002), which also remained significant in the adjusted and fully specified models (all p < .0001). The omnibus main effect of mood on sleep had an effect size of *ω*^*2*^_*p*_ = .042. Higher mood reports were associated with longer reported sleep duration (all p < .0001), with the highest mood level (“+2”) linked to approximately one additional hour of sleep relative to the lowest mood level (“−2”; *β* = 1.00, 95% CI [0.966, 1.029], p < .0001). Hourly sleep-duration patterns differed across mood levels, with more variable time-of-report profiles observed at the extreme mood values (−2 and +2), particularly at the lowest mood level, and more stable profiles observed for intermediate mood levels (−1, 0, and +1; Fig 5).

In addition to this time-of-report pattern, weekday was a significant main effect in predicting reported sleep duration (F(6, 468,085) = 590.85, p < .0001, *ω*^*2*^ = .007), and this association remained significant in both the adjusted and fully specified models (all p < .0001). In the regression model, coefficients for all days of the week were significant (all p < .0001). Saturday and Sunday were associated with higher-than-average sleep duration (Saturday: *β* = 0.117, 95% CI [0.109, 0.126]; Sunday: *β* = 0.190, 95% CI [0.181, 0.198]), whereas weekdays were associated with lower-than-average sleep duration, with the lowest estimates observed on Thursday (*β* = −0.080, 95% CI [−0.088, −0.072]). Overall, reported sleep duration was higher on weekends and lower on weekdays, with Monday showing a modest deviation from this pattern, reflected by a marginally higher coefficient relative to other weekdays (Fig 2).

**Fig 6:**
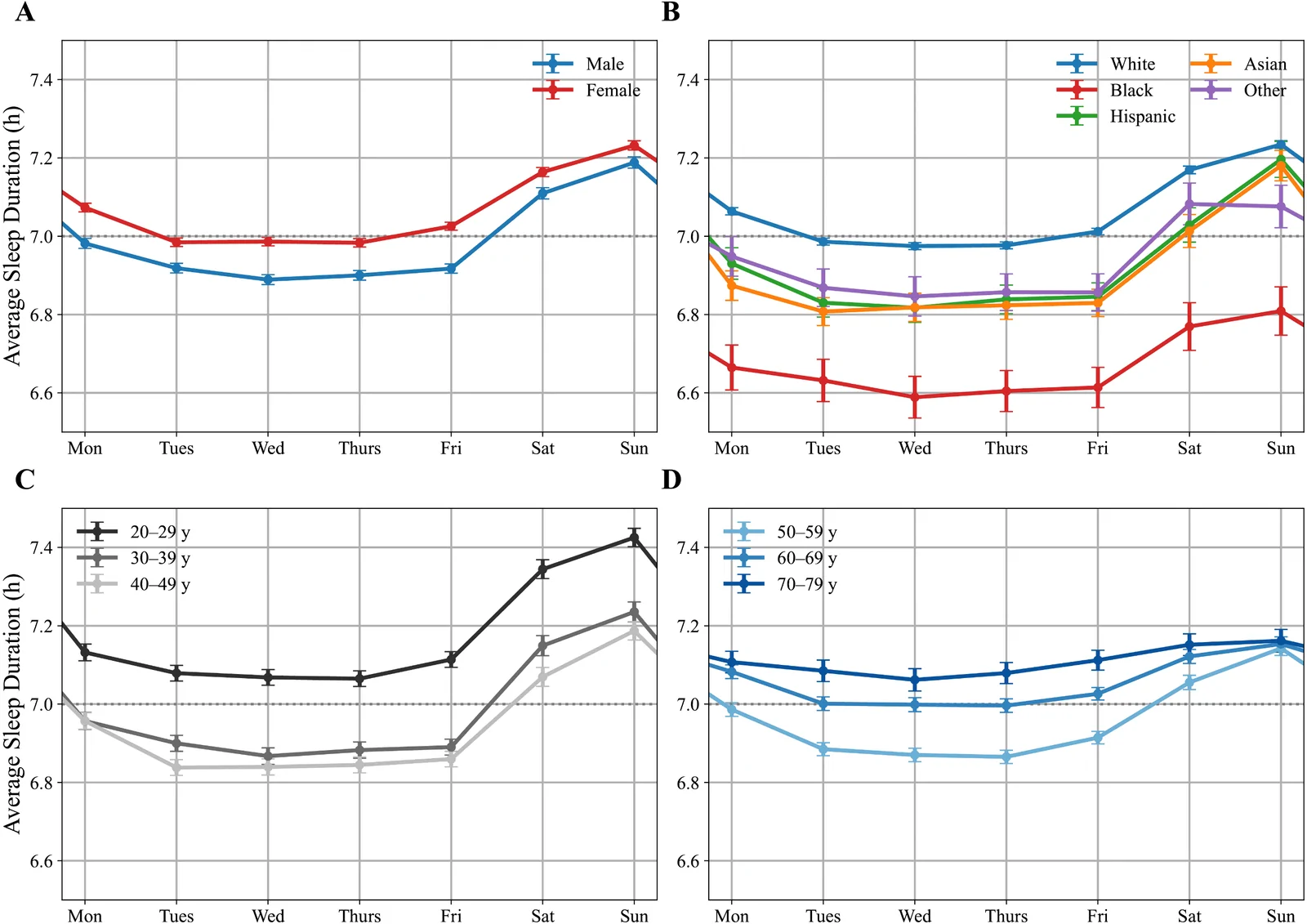
Gender, race/ethnicity, and age variation in average daily reports of sleep duration. (A) Daily sleep duration reports averaged across all hours, plotted for each day and separated by gender. Female users reported higher sleep durations than male users across all days, with the largest difference on weekdays. Sleep duration was higher on weekends than weekdays for both groups, with a larger weekday-weekend difference among male users. Reports were below 7 hours on weekdays and above 7 hours on weekends for both groups. (B) Daily sleep duration reports averaged across all hours, plotted for each day and separated by race/ethnicity. White users reported the highest sleep duration on every day of the week; Asian, Hispanic, and Other users reported similar, lower sleep durations; Black users reported the lowest sleep durations on every day of the week. Sleep durations were higher on weekends than weekdays across all groups, but Black users’ weekend reports remained below 7 hours. (**C–D**) Daily sleep duration reports averaged across all hours, plotted for each day and separated by age. Users aged 20–29 reported the highest sleep durations on average across days, with progressively lower reports across successive age groups and higher reports again among users aged 70–79. The weekday-weekend difference in reported sleep duration was smaller among older age groups, with users aged 70–79 showing the smallest difference. Users aged 20–29 reported sleep durations above 7 hours on every day; users aged 70–79 and, marginally, users aged 60–69 approached this threshold; all other age groups reported sleep durations below 7 hours on weekdays.

To assess whether weekday differences in sleep duration varied across subgroups, two-way ANOVAs were conducted to examine interactions between weekday and gender, race/ethnicity, age, and mood in predicting reported sleep duration (Table 2). A significant interaction was observed between gender and weekday (F(6, 468,078) = 7.36, p < .0001, *ω*^*2*^_*p*_ = 8.15 × 10^-5^), and this interaction remained significant in both the adjusted and fully specified models (all p < .0001). Overall, female users reported longer sleep duration than male users (*β* = 0.079, 95% CI [0.073, 0.086], p < .0001). Across weekdays, male users exhibited lower reported sleep duration relative to female users, whereas female users showed consistently higher sleep duration across the week (Fig 6).

In parallel with gender differences, a significant interaction between race/ethnicity and weekday was observed in predicting sleep duration (F(24, 468,057) = 2.81, p < .0001, *ω*^*2*^_*p*_ = 9.27 × 10^-5^), and this interaction remained significant in both the adjusted and fully specified models (all p < .0001). Across the week, White users reported the highest overall sleep duration, whereas Black users reported the lowest (*β* = −0.390, 95% CI [−0.410, −0.370], p < .0001).

Age further moderated weekday-related variation in sleep duration, with a significant interaction observed between age and weekday (F(30, 468,050) = 1.79, p < .0001, *ω*^*2*^_*p*_ = .001), which remained significant in the adjusted and fully specified models (all p < .0001). Weekday-weekend differences in sleep duration were more pronounced among younger age groups, whereas older age groups (particularly users aged 70–79) exhibited smaller weekday-related variation in reported sleep duration.

By contrast, no significant interaction was observed between mood and weekday in predicting sleep duration (F(24, 468,057) = 1.49, p = .059, *ω*^*2*^_*p*_ = 2.50 × 10^-5^). However, the main effect of mood on predicting sleep duration retained similar magnitude effect sizes as the vice-versa relationship previously described above, *ω*^*2*^_*p*_ = .043.

## Discussion

This study leveraged a large, naturalistic dataset to examine how self-reported mood and sleep duration vary across hour of day, day of week, and demographic group. The primary contribution is not the discovery of hourly mood variation, time-of-report differences in sleep duration, or demographic variation in isolation, as each has precedent in laboratory, epidemiological, or digital-behavioral research. Rather, the contribution is the integration of these patterns within a single, large-scale dataset based on direct user reports. This allows laboratory findings on circadian mood variation, epidemiological findings on sleep duration and demographic disparities, and digital studies of online affective behavior to be explored together. The resulting patterns suggest that mood and sleep are shaped simultaneously by biological timing and social structure: low mood concentrates during the circadian night and morning, sleep duration varies across the workweek, and these patterns differ by age, gender, and race/ethnicity.

Before interpreting these patterns in detail, it is worth addressing the practical magnitude of the effects reported below. Given the scale of this dataset, nearly every main effect and interaction tested reached statistical significance. In samples of this size, significance is only weakly informative about practical importance. To provide a clearer basis for interpretation, we reported omega-squared (*ω^2^*) or partial omega-squared (*ω^2^*_*p*_) for each effect. By conventional benchmarks, most of these effects, including hour of day, weekday, demographic factors, and their interactions, correspond to small effect sizes, with several falling well below the threshold conventionally considered as “small.” These effect sizes indicate that hour of day, weekday, and demographic group membership individually account for only a small fraction of the total variability in any given person’s mood or sleep report. By contrast, the association between mood and sleep duration themselves was substantially larger in magnitude (between .04 and .05) than any temporal or demographic effect reported here. This contrast suggests that, while time-of-day, weekday, and demographic patterns in mood and sleep duration are statistically robust and informative, particularly for understanding the population-level structure and disparities in different social groups, the mood-sleep relationship itself is the most practically substantial association identified in this study.

### Hour-, Weekend-, Age-, Gender-, and sleep-dependent effects on mood

Hourly variation in reports of subjective mood was evident among Lumosity users, with mood peaking midday and reaching its lowest levels overnight. Mood reports were lowest among users logging in during the night, coinciding with the typical sleep period, and higher among users logging in in the morning; average mood reports were highest among users logging in during the afternoon and lower again among those accessing the site in the evening. These temporal patterns closely mirror those documented in laboratory-based circadian and sleep-deprivation experiments [2]. The overnight peak in negative affect is of particular clinical relevance, as it may represent one of several factors contributing to elevated suicide risk during the circadian night [51–54]. Consistent with this literature, individuals who remain awake during the night may experience the full expression of peak negative affect at a time when most people are asleep [2], though the present cross-sectional design cannot confirm this within-person process. It is also important to note that Lumosity users logging in during these hours are a nonclinical, self-selected population engaging with a cognitive-training app, not individuals identified through clinical screening as being at elevated suicide risk. The convergence between our overnight mood findings and the suicide-risk literature is therefore suggestive rather than confirmatory; we suggest that the general pattern of nocturnal negative affect observed is consistent with mechanisms proposed in previous research, but it does not establish that users in our sample who report low mood overnight are experiencing clinically significant risk. Sleep has also been proposed to serve a restorative function that attenuates negative affect; if this mechanism holds, nocturnal wakefulness would be expected to interfere with it, though this cannot be tested directly with the present cross-sectional data. Consistent with these interpretations, analyses of online behavior have identified time-of-day variation in suicide-related content, with post volume on forums such as Reddit’s r/SuicideWatch peaking between 2:00 and 5:00 AM, a window closely aligned with the interval during which Lumosity users reported maximal negative affect and minimal positive affect [55].

Importantly, the present data on mood variation are not unique to our cross-sectional Lumosity user data. Hourly variation in both positive and negative affect has now been identified using multiple online data sources, including self-report measures and linguistic analyses of digital content. Twitter has been extensively used as a social media research tool due to its ability to capture affective states within large, naturalistic contexts. Linguistic coding approaches, most notably the Linguistic Inquiry and Word Count (LIWC), have consistently revealed circadian patterning in emotional expression [56]. Complementary evidence has emerged from other platforms as well. For example, analyses of Spotify listening behavior have shown increased preference for lower-valence, more melancholic music during nighttime and early morning hours, between approximately 23:00 and 06:00 [57]. These time trends closely resemble those observed in Lumosity mood reports, Twitter affective language, and suicide-related forum activity. However, not all online expressions of mood follow this same pattern: emoji usage has been shown to exhibit distinct temporal dynamics, suggesting that visual, textual, and behavioral expressions of affect may reflect partially dissociable processes [58].

Beyond hourly variation, mood reports were higher on Friday than earlier weekdays, highest among users reporting over the weekend, and lower again among those reporting Sunday evening into Monday morning. This cyclical pattern has been widely attributed to increased autonomy, greater social engagement, and temporary relief from occupational stressors during weekends [59–61]. The ability to detect this effect at scale in a large, naturalistic dataset underscores both its robustness across populations and the utility of digital behavioral data for capturing real-world psychological patterns across multiple temporal scales. It is worth noting that, because weighted effect coding expresses each day’s coefficient relative to the weighted average across the full week, the appearance of lower mood on weekdays, and particularly around the start of the week, is partly a function of this comparison structure: when weekend mood is elevated, adjacent weekdays will appear relatively lower even absent any day-specific effect. The overall weekly pattern (elevated weekend mood, lower weekday mood) is therefore a robust and substantive finding, but the relative magnitude of any single weekday’s coefficient, including Monday’s, should be interpreted with this coding structure in mind rather than as fully independent evidence of a day-specific effect.

Across the lifespan, the observed main effect of age on mood reports closely parallels the well-established U-shaped pattern of subjective well-being across age groups. Mood was progressively lower across age groups from young adulthood through midlife, reaching its lowest average in the 40s–50s, and higher again among older age groups. It is worth noting that this pattern reflects a main effect of age on mood rather than a moderating effect of age on weekly mood rhythms: although an age-by-weekday interaction was significant in the unadjusted and partially adjusted models, it did not remain significant after adjustment for sleep duration in the fully specified model. Given the size of the present sample, this suggests the interaction was not robust, and we interpret age as shaping overall mood level rather than the shape of weekly mood variation.

Prior work suggests that midlife is often marked by elevated psychological burden related to career demands, financial strain, and caregiving responsibilities, factors that collectively contribute to lower well-being during this period [62,63]. In contrast, improvements in mood observed in older adulthood have been attributed to enhanced emotional regulation, shifts toward more selective and meaningful social engagement, and reduced exposure to work-related stressors [64]. Notably, our findings also point to greater hour-to-hour variability in reported mood across users in older age groups. This heightened variability may reflect age-related changes in sleep architecture and circadian regulation, such as greater sleep fragmentation and increased sensitivity to circadian disruption, both of which have been linked to within-person mood instability in aging populations [65]. While our between-user comparisons cannot test this mechanism directly, the association between age and hour-to-hour mood variability observed here is consistent with it.

Gender differences further modulated these temporal mood patterns. Although both male and female users reported higher mood on weekends relative to weekdays, female users reported lower mood in the morning compared with male users, despite reporting longer sleep duration on average. This dissociation is consistent with evidence indicating that women experience higher rates of sleep disturbance despite longer self-reported sleep duration, a pattern that may contribute to less favorable mood upon waking [66], though the present cross-sectional data cannot speak to this within-person sequence. At the same time, these findings complement prior laboratory and clinical studies suggesting that women may be particularly sensitive to the mood-disrupting effects of sleep loss [67–69]. While gender differences in sleep and affect have been extensively examined in controlled settings, they remain comparatively underexplored in population-scale, naturalistic datasets such as those provided by Lumos Labs. As such, these results highlight the need for further research to clarify how gender shapes the sleep-mood relationship in everyday contexts.

Finally, sleep duration emerged as a central predictor of mood across temporal scales. Longer sleep duration was associated with more favorable mood, whereas shorter sleep duration was linked to greater hour-to-hour variability in reported mood across users, consistent with prior evidence connecting sleep deprivation to affective dysregulation [70]. Notably, mood reports were lower among users reporting 9 hours of sleep compared with those reporting 8 hours, suggesting that the sleep-mood relationship may not be strictly linear. Excessive sleep duration has been associated with circadian misalignment and increased sleep irregularity, both of which have been linked to poorer mood outcomes [71]. Consistent with the framework outlined above, this sleep-mood association exhibited the largest effect size computed across our analyses (*ω^2^*_*p*_ ≈ .048), underscoring sleep duration as a more substantial correlate of mood than any temporal or demographic factor examined here.

### Hour-, Weekend-, Gender-, Race/Ethnicity-, Age-, and mood-dependent effects on sleep duration

We observed pronounced differences in sleep duration between weekdays and weekends. Across demographic groups, Lumosity users reported longer sleep durations on weekends, consistent with prior longitudinal evidence describing a pattern of weekday sleep restriction followed by longer weekend sleep duration, interpreted in that literature as compensation for accumulated sleep debt [72,73]. Whereas previous studies have typically examined weekday-weekend sleep discrepancies within specific subpopulations, such as adolescents, shift workers, or college students, our findings demonstrate that this pattern extends broadly across the adult lifespan. The consistency of higher weekend sleep durations across groups highlights the pervasive influence of social and occupational constraints on sleep opportunity and sleep health.

Within this broader weekly structure, marked racial and ethnic disparities in sleep duration also emerged. Black Lumosity users consistently reported the shortest sleep durations and showed little difference between weekday and weekend reported sleep duration. Hispanic users likewise reported shorter sleep durations than White users, although their sleep duration exceeded that of Black users. These patterns closely mirror prior research indicating that Black Americans, and increasingly, Hispanic Americans [74], experience chronic sleep restriction, likely driven by socioeconomic and structural factors such as demanding work schedules, neighborhood environments, and chronic stress exposure [75]. Among Hispanic populations, acculturation to U.S. lifestyles has additionally been associated with shorter sleep duration, suggesting another pathway through which sleep disparities may emerge or intensify [76]. We note that these comparisons span more than a decade between our data collection and the cited literature, a gap we discuss further below. Although much of the existing literature on racial and ethnic sleep inequities has relied on national survey data or smaller cohort studies, the ability to detect similar patterns in a large, naturalistic digital dataset underscores their robustness and reinforces the importance of sleep inequity as a public health concern. However, this apparent replication should be interpreted with caution. For instance, Black Lumosity users in 2013–2014 were a self-selected group of cognitive-training app users, and were likely skewed toward higher education attainment, greater digital access, and stronger health engagement relative to Black Americans broadly. As noted in our Limitations section, this sample skew likely means the disparities observed here understate rather than overstate the true population-level gap. It is also worth noting that, while the omnibus effect size for race/ethnicity was low (*ω^2^*_*p*_ ≈ .004), the raw magnitude of the Black-White sleep duration gap (*β* = -0.390 hours) reflects a predicted difference of over 20 minutes in average nightly sleep, a gap that is arguably meaningful in practical terms.

At the same time, these racial/ethnic differences should not be interpreted as intrinsic group differences in sleep need or sleep biology. Rather, they are better understood as indicators of unequal exposure to social and environmental conditions that constrain sleep opportunity. Prior work has linked sleep disparities to work schedules, neighborhood safety, housing conditions, discrimination, chronic stress, healthcare access, and socioeconomic position [77–85]. The present findings add temporal resolution to this literature by showing that shorter reported sleep among Black users was evident regardless of time-of-report, and across the week, with a smaller weekday-to-weekend difference than that reported by other demographic groups. This pattern is consistent with the possibility that some groups experience not only shorter habitual sleep duration, but also less opportunity for the weekday-to-weekend increase seen in other groups.

Time-of-report and weekly patterns of sleep duration varied systematically across the adult lifespan. Reported sleep duration was progressively lower across successive age groups, with the largest between-group differences observed between ages 30 and 60, and comparatively small differences among groups older than 60, potentially reflecting lifestyle transitions such as retirement and reduced external obligations [36]. The scale of the Lumosity dataset enabled detailed visualization of these age-related differences across the full adult lifespan, highlighting middle adulthood as a particularly vulnerable period for sleep loss.

The age-related findings have social and occupational relevance, although they should be interpreted cautiously. The lowest levels of reported sleep duration (as well as mood) occurred during midlife, a period often characterized by overlapping work, financial, caregiving, and family demands, particularly as adults navigate peak employment years, childrearing or support of young-adult children, care for aging parents, and preparation for later-life financial security [86–88]. These data cannot identify which demands were responsible, because employment status, caregiving burden, shift work, and socioeconomic position were not measured by Lumosity. Nevertheless, the pattern is consistent with the view that midlife sleep may be shaped strongly by external schedule constraints. Workplace policies, schedule flexibility, and sleep-supportive occupational practices may therefore represent relevant downstream implications of the present analyses, particularly given evidence that schedule predictability, work-time control, and workplace-based interventions can influence sleep opportunity, sleep duration, and sleep quality [89–92].

Finally, sleep duration was closely coupled with affective state. Higher mood was associated with longer sleep duration, whereas users reporting low mood exhibited greater variability in reported sleep duration across hours of report, reflecting a more variable hourly profile. This is consistent with prior work linking sleep deprivation to heightened negative affect and affective dysregulation [70]. The scale and temporal resolution of the Lumosity dataset allowed these associations to be captured across a large number of naturally occurring report times, reinforcing the close association between sleep and mood in everyday life. As in the mood models described above, this association was the largest effect size observed in our analyses of sleep duration models (*ω^2^*_*p*_ ≈ .042 to .043), further highlighting mood as a stronger correlate of sleep duration than any temporal or demographic variable considered.

### Limitations

Several limitations should be considered when interpreting these findings. First, the Lumosity mood and sleep items were brief app-based self-report measures rather than standardized clinical instruments or objective physiological assessments. The five-point mood item captured broad affective valence at the time of login, but it could not distinguish among specific states such as sadness, anxiety, irritability, stress, or anhedonia, nor can it be interpreted as a diagnostic measure of depression or other mood disorders. Similarly, the sleep item captured binned self-reported sleep duration from the prior night, but did not assess sleep onset, wake time, sleep efficiency, nighttime awakenings, sleep quality, napping, circadian phase, or sleep architecture. The sleep-duration variable also had important statistical constraints. Responses were collected in discrete bins, with open-ended lower and upper categories of ≤5 and 9 + hours that were coded as 5 and 9, respectively, for analysis. As a result, the measure compressed variation at the extremes of short and long sleep duration and could not distinguish, for example, 3 hours from 5 hours or 9 hours from 11 hours. Treating this variable as an interval outcome therefore provided a practical approximation for large-scale modeling, but it may have attenuated associations, obscured nonlinear effects, and limited interpretation of findings at the tails of the sleep-duration distribution. Thus, the findings should be interpreted as large-scale patterns in brief self-reported mood and coarsely binned sleep-duration estimates, not as evidence concerning objectively measured sleep or clinically validated mood states.

Second, the dataset was cross-sectional and included only one record per user. Accordingly, the observed associations between mood, sleep duration, hour of day, weekday, and demographic characteristics cannot establish directionality or within-person change. Hourly and weekly patterns should therefore be understood as differences among users who logged in at different times, rather than as repeated observations of the same individuals across the day or week.

Third, the voluntary, app-based nature of the sample limits generalizability. Lumosity users may differ from the broader population in digital access, education, health status, interest in cognitive training, employment patterns, and other factors relevant to sleep and mood. Although the sample was large and age-diverse, it was disproportionately composed of White, female, and highly educated users (S1 Table). In addition, users aged 15–19 and 80–90 years, as well as users identifying as Native American or Pacific Islander, were excluded from the primary subgroup analyses because of sparse or incomplete representation across the temporal strata required for hour-of-day and weekday models. As a result, the findings should not be generalized to adolescents, adults over 80, Native American users, Pacific Islander users, or other underrepresented groups without further study.

Race/ethnicity analyses warrant particular caution. The categories available in the Lumosity dataset combined racial and ethnic classifications and did not permit more granular characterization of ancestry, culture, immigration history, socioeconomic position, or structural exposures. Some groups were also underrepresented across hour-of-day and weekday strata, increasing the risk of unstable subgroup estimates. These analyses are therefore best understood as exploratory descriptions of group-level differences in self-reported sleep duration within this platform, rather than as mechanistic explanations of racial or ethnic disparities. Moreover, because participation required both digital access and sustained engagement with a cognitive-training platform, users identifying as Black or Hispanic in this sample may not be representative of Black or Hispanic Americans broadly and may be skewed toward higher socioeconomic status and health engagement. This pattern would likely attenuate, rather than inflate, observed disparities relative to the general population.

A related but distinct limitation concerns the age of the dataset itself. These data were collected in 2013–2014 and are well over a decade at the time of this report. In the years since, smartphone and wellness-app adoption has expanded substantially and become more demographically diverse, potentially altering who uses platforms like Lumosity relative to a decade ago. Broader shifts in sleep patterns and public discourse around mental health, including changes associated with the COVID-19 pandemic, may also limit the extent to which these findings reflect contemporary populations. Some patterns reported here are likely more robust to this gap than others. The hourly and weekly mood and sleep patterns observed closely mirror findings from decades of laboratory-based circadian research, suggesting these core temporal rhythms are unlikely to have changed substantially. By contrast, the demographic disparity findings, particularly those of race/ethnicity and digital access, may be more vulnerable to these temporal drifts, given that the composition of digital health-app users may have shifted since 2013–2014. Replication using more recent datasets, ideally collected across multiple digital health platforms including and beyond Lumosity, will be necessary to determine whether these patterns persist in contemporary populations.

A further limitation concerns the login-based nature of data collection. Reports were initiated when users accessed Lumosity rather than being prompted at randomly assigned or experimentally scheduled times. As a result, hour-of-day patterns may partly reflect differences in who logs into the platform at particular times (though the analyses controlled for this possibility to the extent that they could). For mood, login hour provides a reasonable timestamp for current affective state, although users awake and using Lumosity overnight may differ systematically from daytime users. This issue is especially important for sleep duration. Because the sleep item asked users to report how many hours they slept “last night,” hour of day indexes the time of report rather than the timing, midpoint, or duration of the sleep episode itself. Users logging in later in the day may include individuals with later wake times, greater schedule flexibility, delayed sleep timing, shift-work schedules, or other unconventional sleep patterns. Because the dataset did not include chronotype, employment status, work schedule, bedtime, wake time, sleep regularity, or comorbid mental health conditions, we cannot determine the extent to which these factors contributed to the observed patterns. Accordingly, hourly sleep-duration findings should be interpreted as descriptive time-of-report patterns in self-reported prior-night sleep, not as evidence of endogenous diurnal rhythms in sleep duration.

Future studies incorporating longitudinal designs, externally prompted assessments, wearable or diary-based sleep measures, ecological momentary affect ratings, and targeted recruitment of underrepresented groups will be needed to determine whether these temporal patterns generalize beyond Lumosity users and to clarify the mechanisms underlying the observed associations.

## Conclusion and implications

The present findings carry potential public health significance because they show that mood and sleep duration vary together across multiple timescales: hour of day, day of week, and stage of adulthood. These patterns suggest that sleep and mood vulnerability are not randomly distributed across the population or across time, but instead emerge at the intersection of biological timing, social schedules, and demographic context. In this respect, the primary contribution of the present study is the ability to replicate experimental findings at scale, using self-reports from approximately 468,000 users collected in a naturalistic, digital environment.

One implication concerns midlife as a potential period of heightened sleep and mood vulnerability. In the present temporal analyses, users in their 30s through 60s showed lower sleep duration and less favorable mood than younger and older adults. The significance of this pattern is not simply that midlife users reported worse sleep or mood, but that these two domains converged during a life stage that prior life-course research links to later health outcomes. Midlife is increasingly understood as a pivotal period in the life course, linking earlier exposures to later-life outcomes and offering an opportunity for prevention before sleep disturbance, affective burden, and related health risks become more entrenched. From this perspective, the present findings suggest that large-scale digital platforms may be useful for generating hypotheses about life stages in which sleep and mood vulnerability could jointly emerge, pending confirmation in more representative and controlled follow-ups. Such signals are not diagnostic, nor do they identify the mechanisms responsible, but they may help guide future longitudinal studies aimed at determining whether midlife sleep-mood profiles predict later mental health, cognitive, or cardiometabolic outcomes. Thus, the public health relevance of the age-related findings lies less in any single policy prescription than in highlighting midlife as a potentially informative window for detection, prevention, and follow-up.

A second implication concerns the convergence of nighttime negative affect and elevated overnight suicide risk. Across analyses, mood followed a robust hourly pattern, with the lowest reports occurring between 0:00 and 06:00. This interval aligns with prior evidence linking the circadian night to heightened rumination, impaired psychological regulation, and increased suicidality [51,53,93]. The importance of this finding lies in its temporal specificity. Many mental health services are less available during the overnight hours [94], yet this is precisely the interval during which negative affect may intensify among individuals who are awake. The present findings are consistent with the view that the circadian night is a clinically important window of vulnerability, especially for individuals with insomnia, depression, mood disorders, or nocturnal distress. It is important to be clear about the limits of this inference, however: our sample consists of nonclinical users within a cognitive-training app, not individuals identified through clinical screening as being at elevated suicide risk, and their mood ratings cannot be used to directly estimate suicide risk or crisis-service need. Rather than providing direct evidence for expanded overnight crisis capacity, our findings offer converging, population-level support for a pattern established in the clinical and epidemiological literature: that negative affect and psychological distress tend to intensify during the circadian night, a period when mental health services are less available. Considered alongside that literature, this pattern strengthens the rationale for further research into nighttime mental health support and screening, though the present data alone cannot establish the specific policy or clinical interventions warranted [95].

Another implication concerns sleep inequity. The replication of racial-ethnic disparities in reported sleep duration indicates that patterns previously documented in epidemiological research are also visible in a large naturalistic digital sample. Black and Hispanic users reported shorter sleep durations than White users, and Black users showed the smallest weekday-to-weekend increase in reported sleep duration. This pattern is important because weekend sleep extension is consistent with a recovery process, in which individuals compensate, at least partially, for sleep restriction accumulated during the workweek. However, the present cross-sectional data cannot confirm this within-person mechanism. The smaller increase in reported sleep duration among Black users, if it does reflect reduced opportunity for recovery, suggests that sleep inequity may involve not only shorter habitual sleep duration, but also fewer opportunities to compensate for sleep loss when it occurs. In this sense, sleep disparities may reflect differences in sleep opportunity, schedule control, environmental burden, and exposure to chronic stress, rather than differences in sleep need. The present findings add temporal resolution to the sleep-disparities literature by showing that unequal sleep duration was evident not only as an overall group difference, but also in the weekly pattern of weekday / weekend sleep. Addressing sleep inequity will likely require interventions that extend beyond individual sleep hygiene advice, which assumes that sleep behavior is primarily under personal control. Instead, meaningful improvement may require structural and policy-level efforts that increase the conditions under which healthy sleep is possible, including more predictable work schedules, safer and quieter sleep environments, and improved access to preventive sleep and mental health care. Of course, these conclusions should be considered alongside the nature of the sample: Black and Hispanic users in this sample were likely a more advantaged subset of their respective populations, which suggests the true population-level disparity is probably larger than these estimates indicate, strengthening rather than weakening the case for structural intervention.

Beyond these practical applications, our study ultimately demonstrates the power of large-scale digital datasets in replicating and extending findings from traditional sleep and circadian research. The ability to observe well-established mood and sleep patterns within a single, naturalistic dataset supports the external validity of prior findings and highlights the unique value of digital behavioral data in psychological and health research. Future studies should expand big data approaches by integrating wearable sleep tracking, passive sensing technologies, and ecological momentary assessments to provide even deeper insights into real-world sleep and mood dynamics. Given that the present data were collected over a decade ago, future work using more recent data will also be important for confirming whether these patterns, particularly the demographic disparities observed here, persist in contemporary populations.

## Supporting information

S1 Table Descriptive statistics of raw and analyzed samples. S1A shows the raw sample prior to decade-based age grouping and exclusion of underrepresented demographic categories. S1B shows the analytic sample used in all primary analyses.
(PDF)
