# Supplementary material for: Large-scale patterns of self-reported mood and sleep across the day and week on an online cognitive-training platform
Source: PLOS Digit Health. 2026 Sep 24;5(9):e0001754. doi: 10.1371/journal.pdig.0001754 (PMC13606931; doi:10.1371/journal.pdig.0001754)
Supplement: Supplementary file 1 [file pdig.0001754.s001.pdf]

Table S1A. Descriptive Statistics of Raw Sample (N = 493,758)

Variable	Category	n	%
**Gender**			
	Male	284,262	42.4
	Female	209,496	57.6
**Age**			
	15-19	13,106	2.7
	20-24	38,867	7.9
	25-29	47,229	9.6
	30-34	39,847	8.1
	35-39	30,505	6.2
	40-44	32,379	6.6
	45-49	37,862	7.7
	50-54	50,481	10.2
	55-59	55,164	11.2
	60-64	54,762	11.1
	65-69	46,085	9.3
	70-74	27,508	5.6
	75-79	12,895	2.6
	80-84	5,349	1.1
	85-90	1,719	0.3
**Race / Ethnicity**			
	White	409,925	83.0
	Black	12,292	2.5
	Hispanic	23,866	4.8
	Asian	26,615	5.4
	Other	15,218	3.1
	Native American	4,051	0.8
	Pacific Islander	1,791	0.4
**Mood**			
	-2	6,496	1.3
	-1	40,902	8.3
	0	209,322	42.4
	1	214,384	43.4
	2	22,654	4.6
**Sleep Duration**			
	≤5	47,880	9.7
	6	109,479	22.2
	7	157,580	31.9
	8	135,513	27.4
	9+	43,306	8.8

Table S1B. Descriptive Statistics of Analyzed Sample (N = 468,092)

Variable	Category	n	%
**Gender**			
	Male	196,360	41.9
	Female	271,732	58.1
**Age**			
	20-29	84,770	18.1
	30-39	69,375	14.8
	40-49	69,387	14.8
	50-59	104,485	22.3
	60-69	99,971	21.4
	70-79	40,104	8.6
**Race / Ethnicity**			
	White	394,450	84.3
	Black	22,617	4.8
	Hispanic	11,698	2.5
	Asian	24,883	5.3
	Other	14,444	3.1
**Mood**			
	-2	6,086	1.3
	-1	38,919	8.3
	0	198,858	42.5
	1	203,302	43.4
	2	20,927	4.5
**Sleep Duration**			
	≤5	45,257	9.7
	6	104,659	22.4
	7	150,644	32.2
	8	128,286	27.4
	9+	39,246	8.4
