# Supplementary material for: Large-scale patterns of self-reported mood and sleep across the day and week on an online cognitive-training platform
Source: PLOS Digit Health. 2026 Sep 24;5(9):e0001754. doi: 10.1371/journal.pdig.0001754 (PMC13606931; doi:10.1371/journal.pdig.0001754)
Supplement: Supplementary file 2 [file pdig.0001754.s002.pdf]

DEPARTMENT OF PSYCHOLOGY
Life Sciences North, Room 349
1501 N. Campbell Avenue
Tucson, AZ 85724

Ofc: 520-626-2489
EMAIL: ramendoza@arizona.edu

THE UNIVERSITY OF ARIZONA
College of Science

May 23rd, 2026

Girum Tareke Zewude, PhD
Academic Editor
PLoS Digital Health

RE: Resubmission of Ms. No. PDIG-D-26-00336

Dear Dr. Zewude:

Thank you for considering our manuscript for publication in *PLOS Digital Health* and for the opportunity to reply to the comments from the reviewers. We would like to thank the reviewers for their kind words regarding the significance and quality of our manuscript. Moreover, we appreciate the fact that the reviewers took the time to detail the sorts of clarifications and added information that will serve to strengthen our paper. We want to assure you that we have taken these comments to heart and, in response, have rewritten parts of the manuscript per their guidance. Our point-by-point responses are presented below with the reviewer's original comments in *italics*. As presently written, we hope that you will agree that the manuscript represents a valuable contribution to the journal that will stimulate further work and discussion on the intersections between sleep and mood in large naturalistic datasets.

Sincerely,

Reina A. Mendoza, MA

DEPARTMENT OF PSYCHOLOGY
Life Sciences North, Room 349
1501 N. Campbell Avenue
Tucson, AZ 85724

Ofc: 520-626-2489
EMAIL: ramendoza@arizona.edu

THE UNIVERSITY OF ARIZONA
College of Science

Comments to the Author

Reviewer 1

1a. *"The rationale for excluding certain age groups (15–19 and 80–90) and specific ethnic categories such as Native Americans and Pacific Islanders is not fully justified. The authors should explain whether these exclusions were based on sample size limitations or potential biases, and discuss the implications for generalizability of the findings. Without this clarification, it is difficult to assess how representative the sample is of the broader population."*

Response: We agree that the original manuscript did not adequately justify the exclusion of the 15–19 and 80–90 age ranges or the Native American and Pacific Islander race/ethnicity categories. We have revised the Methods and Limitations sections to clarify that these exclusions were based on sample-size and modeling considerations rather than on theoretical or substantive judgments about the relevance of these groups (all additions here and elsewhere have been marked in green text).

Specifically, the study was designed to examine hour-of-day and day-of-week patterns across demographic subgroups. Because these analyses require observations distributed across many temporal strata, relatively small demographic categories can yield unstable estimates, wide uncertainty, and potentially misleading subgroup patterns. The 15–19 and 80–90 age ranges were also incomplete edge categories relative to the decade-based adult bins used in the primary analyses. For this reason, we restricted the primary age analyses to full adult decade bins from 20–29 through 70–79 years. Similarly, Native American and Pacific Islander users were not included in subgroup-specific race/ethnicity models because sparse representation across hour-of-day and weekday cells raised concerns about unstable estimates and overinterpretation.

We have also revised the manuscript to discuss the implications of these exclusions for generalizability. The findings should be interpreted as applying most directly to Lumosity users aged 20–79 in the analyzed race/ethnicity categories, and not as representative of adolescents, adults over 80, Native American users, Pacific Islander users, or the broader population. We now make clear that these exclusions may reduce representativeness and that future studies with targeted recruitment or larger subgroup samples are needed to characterize sleep and mood rhythms in these underrepresented groups.

1b. *"The measurement of mood and sleep duration, while described, lacks sufficient information on validation. The five-point mood scale and the binned sleep duration categories may not fully capture the variability in affective states or sleep patterns. The authors should provide evidence that these self-*

DEPARTMENT OF PSYCHOLOGY
Life Sciences North, Room 349
1501 N. Campbell Avenue
Tucson, AZ 85724

Ofc: 520-626-2489
EMAIL: ramendoza@arizona.edu

report measures are reliable and, if possible, validated against objective data such as actigraphy or clinical assessments. This would strengthen confidence in the accuracy of the outcomes analyzed."

Response: We agree that the original manuscript described the mood and sleep-duration measures but did not sufficiently discuss their measurement limitations or validation status. In the revised manuscript, we have expanded the Methods and Limitations sections to clarify that the Lumosity mood and sleep items were brief, app-based self-report measures rather than clinical assessments or objective sleep measures.

Specifically, we now state more clearly that the five-point mood item should be interpreted as a broad measure of momentary affective valence, not as a validated measure of positive affect, negative affect, depression, anxiety, or clinical mood state. Similarly, the sleep-duration item should be interpreted as a coarse self-report estimate of the prior night's sleep duration, not as a measure of sleep architecture, sleep quality, sleep timing, insomnia symptoms, or objectively measured sleep. We have also clarified that the binned sleep categories limit precision, especially at the lower and upper ends of the range.

We were not able to validate these measures against actigraphy, polysomnography, sleep diaries, clinical interviews, or standardized affective inventories in the present dataset. Accordingly, we have revised the manuscript to avoid implying that the outcomes are equivalent to objective or clinically validated measures. Instead, we frame the strength of the dataset as its large scale, naturalistic context, and direct user self-report, while recognizing that this comes at the cost of reduced psychometric and physiological precision.

1c. *"The manuscript mentions rounding the extreme sleep categories (≤5 hours and 9+ hours) to 5 and 9, but does not discuss how this transformation might influence statistical analyses. The authors should elaborate on potential effects on variance, regression coefficients, and interpretation of extreme sleep durations, particularly since these extremes may carry important behavioral or clinical implications."*

Response: We agree that rounding the categories of ≤5 hours and 9+ hours has the potential to influence statistical analyses. Lumosity's survey prompts only provided singular hour responses for 6, 7, and 8 hours for sleep duration. For the extreme ends, a user would see the choices "≤5" and "9+" and not be able to specify it beyond that spectrum. Our revised manuscript has clarified this detail in our methods. We have also revised our limitations section to provide insight on how this decision may lead to attenuated associations, obscured nonlinear effects, and limited interpretation at the tails of the sleep-duration distribution.

DEPARTMENT OF PSYCHOLOGY
Life Sciences North, Room 349
1501 N. Campbell Avenue
Tucson, AZ 85724

Ofc: 520-626-2489
EMAIL: ramendoza@arizona.edu

THE UNIVERSITY OF ARIZONA
College of Science

1d. *"The description of regression models (Models 1–3) is somewhat confusing and dense. While the distinction between unadjusted, adjusted, and fully specified models is noted, a clearer presentation is needed. Including a summary table or diagram that shows the main effect, covariates, complementary outcome, and interaction terms for each model would enhance readability and allow readers to follow the statistical strategy more easily."*

Response: We agree that our three-model structure needed further explanation. In the revised manuscript, we have added a comprehensive diagram explaining how the models are implemented (**Fig 1**), and the two results tables have been visually and verbally tweaked to provide better descriptions of the data.

1e. *"Weighted effect coding is applied for hour and weekday variables, but the rationale for choosing this method over standard dummy coding is not fully explained. The authors should justify why weighted effect coding was selected and discuss how it influences the interpretation of regression coefficients compared to unweighted or reference-based coding methods. This will help clarify the analytical decisions for the reader."*

Response: We have now clarified our rationale for using weighted effect coding in the Methods section. We did not apply dummy coding, which requires identifying a reference category, because it reflects the hypothesis that one clock hour or weekday has higher (or lower) sleep or mood ratings than another day. By contrast, we merely hypothesized that there would be variability within the day or across days, which suggests a null hypothesis of no within-day or across-day variation.

1f. *"The modeling of interactions, especially between hour/weekday and demographic or behavioral factors, is central to the analysis. However, the criteria for including interaction terms in Models 2 and 3 are not clearly described. The authors should specify whether only interactions significant in Model 1 were carried forward, and whether any adjustments for multiple comparisons were applied. Clearer documentation of the interaction modeling strategy would improve reproducibility and interpretability."*

Response: The revised analysis section has clarified how the pipeline between Models 1, 2, and 3 operate (i.e., the next model is performed only if the previous model has a significant effect). Our diagram in the newly added **Fig 1** also displays this workflow visually and makes the sequence easier to follow. As for multiple comparisons, we have now described our approach after determining significance across all three models. Specifically, regression coefficients were extracted from Model 1 only if the main effects and interaction terms retained significance across all three ANOVA models. Models 2 and 3 are used to check if main effect or interaction terms retain significance after adjusting

DEPARTMENT OF PSYCHOLOGY
Life Sciences North, Room 349
1501 N. Campbell Avenue
Tucson, AZ 85724

Ofc: 520-626-2489
EMAIL: ramendoza@arizona.edu

THE UNIVERSITY OF ARIZONA
College of Science

for extra factors. Because coefficients were extracted from a single omnibus model (Model 1) rather than from a separate post-hoc test of the same outcome groupings, no additional correction for multiple comparisons was applied.

1g. *"The use of one-way and two-way ANOVAs to assess main effects and interactions requires additional justification. Given that mood and sleep measures are ordinal or interval variables, the authors should clarify why ANOVA is appropriate and whether assumptions such as normality and homogeneity of variance were tested. Addressing these points will strengthen the methodological rigor of the statistical analyses."*

Response: Thank you for raising this important methodological point. Our revised manuscript has clarified the use of ANOVA through several points below:

While mood and sleep duration are measured on ordinal scales (-2 to +2 for mood; 5 to 9 for sleep duration), treating them as interval is common and defensible in large-scale psychology research, particularly with symmetric Likert scales of approximately equal-interval interpretations (Norman, 2010; Sullivan & Artino, 2013). We acknowledge it is difficult to run assumption checks with discrete data using QQ-plots (for normality) and box plots (for homogeneity). However, our sample size provides substantial robustness against these assumption checks. For the assumption of normality, our massive sample size (nearly 500,000) is supported by the Central Limit Theorem, where the sampling distribution of the mean approaches normality regardless of the population distribution. Because large sample sizes are extremely sensitive to statistical tests of homogeneity such as Levene's test, to test whether this assumption violation would impact results, we performed Welch's ANOVA tests (for data not assuming homogeneity) on our one-way models (since you cannot run Welch on interaction terms) and found they yielded similar statistics and significance values to our Type III ANOVA tests. All of these considerations are now clarified in detail in the analysis section of the manuscript, which has been heavily revised.

1h. *"The temporal aggregation of data into hourly and weekly bins is not fully described. It is unclear how multiple entries from the same user or uneven login times were handled. The authors should clarify whether data were aggregated per user or pooled across users, and whether weighting was applied to account for unequal contributions. This information is critical for understanding the derivation of diurnal and weekly patterns."*

Response: In our revised manuscript, we clarify that each data point represents one unique user at a given time and day. This means that any user will only appear once in the entire dataset and not across multiple time points or day points. Furthermore, Type III Sum of Squares accounts for unequal sizes,

DEPARTMENT OF PSYCHOLOGY
Life Sciences North, Room 349
1501 N. Campbell Avenue
Tucson, AZ 85724

Ofc: 520-626-2489
EMAIL: ramendoza@arizona.edu

THE UNIVERSITY OF ARIZONA
College of Science

while weighted effect coding ensures coefficient interpretation is relative to the grand average rather than a single reference group.

As for the login times represented, we have now clarified that any given user's login time is rounded from a range of the clock hour encompassing it (e.g., the 23:00 bin included all logins between 23:00 and 23:59). Please see the revised Methods section for further detail.

1i. *"Demographic covariates, including age, gender, and ethnicity, are treated categorically. The authors should provide a rationale for using categorical bins rather than continuous measures, particularly for age. Additionally, potential collinearity among covariates should be assessed and reported, as it could influence the interpretation of regression coefficients and interactions."*

Response: While age could reasonably be treated as a continuous variable, we were mainly interested in age as it pertains to established developmental stage distinctions (young adulthood, middle age, older adulthood, etc.) that would be more theoretically meaningful to our discussion of the dataset. Therefore, we decided to bin the decades and treat them as a categorical variable similar to the rest of the covariates.

As for the potential for collinearity, we have now reported evidence against it through Variance Inflation Factors (VIF). Briefly, the adjusted generalized standard error inflation factors (aGSIF) for all variables of interest returned values between 1.0–1.5, indicating no problematic collinearity between the variables. To keep this test concise and comprehensive, we created two models: one predicting mood with all relevant factors, and one predicting sleep duration with all relevant factors. This includes listing the opposite outcome (mood for the sleep duration model, and vice versa) as categorical factors within the models.

1j. *"Finally, while the manuscript notes that analyses were performed in R and Python, it does not provide sufficient detail for reproducibility. The authors should specify packages, functions, and versions, and ideally provide a reproducible workflow or code repository. This transparency is essential given the complexity of the modeling approach and the large dataset analyzed."*

Response: We agree that having a very reproducible workflow and code repository would greatly aid in the transparency of these findings. Our revised manuscript has detailed the versions of R and Python used as well as the key packages and functions we relied on for data processing, statistical modelling, and visualization. Additionally, we have prepared a "ready-to-publish" repository for the code workflow on Zenodo (doi.org/10.5281/zenodo.20330348) and alternatively on GitHub (raemendoza/lumosity) that would reproduce the statistical results and graphics shown in our manuscript. Although the data are owned by Lumosity, we provided a format example of the data

DEPARTMENT OF PSYCHOLOGY
Life Sciences North, Room 349
1501 N. Campbell Avenue
Tucson, AZ 85724

Ofc: 520-626-2489
EMAIL: ramendoza@arizona.edu

THE UNIVERSITY OF ARIZONA
College of Science

columns within the repository to ensure the code is able to properly run on a prepared dataset. For reviewer purposes, the code is available here: Zenodo Record.

Reviewer 3

3a. *"Unfortunately, I could not get a strong sense of importance of the research questions or findings from the paper. The authors tie their findings to suicide prevention, workplace policies, and socioeconomic disadvantage. These are emotionally powerful and the authors cite relevant works [32-36, 59, 60]. However, the paper could provide more analysis of the literature and deeper explanation to make the importance of the work clearer. The authors also motivate the research citing contradictory findings about sleep architecture and aging. However, that literature is not critically examined."*

Response: We thank the reviewer for this thoughtful critique. We agree that the prior version of the manuscript did not sufficiently develop the importance of the research questions or provide enough conceptual scaffolding for the public health implications we discussed. In the revised manuscript, we have strengthened the Introduction and Discussion in several ways.

First, we now more explicitly frame the study as an attempt to bridge three bodies of evidence that are often treated separately: laboratory circadian studies of mood, epidemiological studies of sleep duration and demographic disparities, and large-scale digital-behavioral studies that infer affect or sleep from online activity. We clarify that the value of the present dataset is not that it replaces laboratory or clinical assessment, but that it provides a large-scale, direct self-report complement to those literatures.

Second, we have more critically examined the aging and sleep literature. The prior version cited apparently contradictory findings regarding age-related changes in sleep architecture and epidemiological patterns of sleep duration but did not sufficiently explain why these literatures may diverge. We now clarify that polysomnography and actigraphy primarily characterize sleep architecture, continuity, and timing, whereas large epidemiological and app-based studies often capture perceived or reported sleep duration. These are related but non-equivalent constructs. Thus, age-related reductions in slow-wave sleep and increased sleep fragmentation can coexist with stable or even longer self-reported sleep duration in later life, particularly if retirement, reduced work demands, or altered sleep opportunity/flexibility change how sleep is experienced and reported. We have revised the manuscript accordingly to make clear that our findings speak most directly to self-reported sleep duration in everyday life, not to objective sleep architecture.

Finally, we have revised the public health implications to be more measured and better supported. Rather than presenting suicide prevention, workplace policy, and socioeconomic disadvantage as broad

DEPARTMENT OF PSYCHOLOGY
Life Sciences North, Room 349
1501 N. Campbell Avenue
Tucson, AZ 85724

Ofc: 520-626-2489
EMAIL: ramendoza@arizona.edu

THE UNIVERSITY OF ARIZONA
College of Science

conclusions, we now frame them as interpretive domains where the observed temporal and demographic patterns may be relevant, while acknowledging the limits of causal inference.

3b. *"I have concerns about the methodology. I could not find some important characteristics of the dataset, such as: (a) what time period the data covers, (b) what were the triggers prompting users to self-report, (c) the numbers of mood and sleep reports, and (d) basic demographic statistics of the dataset. My main methodological concern is that simple things like record numbers or data collection biases may contribute to some findings. The nature of the trigger (b) may introduce significant biases into the analysis. Additionally, for sleep duration data I don't have a clear appreciation of why time-of day has a clean meaning. The authors already draw attention to biases from users self-selecting to be in the study. If data collection was self-triggered this introduces a potentially devastating bias – one that is not measured and not discussed. Additionally, it is not clear to me what effects on findings there may be from shift work and unconventional sleeping habits."*

Response: We thank the reviewer for raising this important methodological concern. We agree that the original manuscript did not provide sufficient descriptive information about the dataset structure, reporting context, and possible sampling biases. We have revised the Methods section to clarify that the data were collected from Lumosity users between 2013 and 2014; that each analytic record corresponds to a unique user sampled once, not repeated observations from the same individual; and that mood and sleep duration were reported when users logged into the platform for a daily brain-training assessment. We have also added descriptive information on the basic demographic characteristics of the raw and analytic sample (**S1 Table**). In addition, we now clarify that hour of day refers to the time of login/report, not objectively measured sleep timing. This distinction is especially important for sleep duration analyses: time-of-day effects in sleep reports should be interpreted as differences in reported prior-night sleep duration among users logging in at different times, not as direct evidence of diurnal variation in sleep itself. Finally, we have expanded the Limitations section to acknowledge that self-triggered login behavior, unmeasured chronotype, employment status, shift work, unconventional sleep schedules, and other user-selection factors may contribute to the observed patterns. Accordingly, we now frame these analyses as descriptive patterns in self-reported mood and sleep among Lumosity users rather than as causal or population-generalizable estimates.

3c. *"I am also a little confused about data on Black, Hispanic, White, etc. Firstly, it is not clear to me what these categories are. E.g., are they race or ethnicity, or some conflation of the two. Also, I could not see the scientific need for considering this variable; there was no associated research question and the only relevant conclusion merely confirmed existing knowledge. Given the questionable value of this variable, it may be better to simply ignore it for analysis. This also eliminates any need to discard categories Native American and Pacific Islanders due to underrepresentation."*

DEPARTMENT OF PSYCHOLOGY
Life Sciences North, Room 349
1501 N. Campbell Avenue
Tucson, AZ 85724

Ofc: 520-626-2489
EMAIL: ramendoza@arizona.edu

THE UNIVERSITY OF ARIZONA
College of Science

Response: We thank the reviewer for raising this important concern. We agree that the prior manuscript did not adequately define the demographic categories or explain why this variable was included. In the revised manuscript, we now refer to this variable as race/ethnicity rather than "ethnicity" alone, because the categories provided in the Lumosity dataset combine socially defined racial and ethnic classifications. We have also clarified that these categories were self-reported or user-provided within the platform and were analyzed as social-demographic descriptors, not as biological or genetic categories.

We also agree that the rationale for including this variable needed to be made more explicit. Our purpose was not simply to confirm already known disparities, but to examine whether previously documented sleep disparities are observable in a large naturalistic digital dataset and whether they vary across hour of day and day of week. This temporal dimension is the specific contribution of the present analysis: prior work has documented racial/ethnic differences in sleep duration, but fewer studies have examined whether such differences are stable across the day and week or whether weekend recovery patterns differ by group. Accordingly, we have revised the Methods and Discussion to frame these analyses as exploratory, descriptive, and grounded in the sleep-health disparities literature.

Finally, we have clarified the treatment of smaller demographic groups. The original analysis excluded Native American and Pacific Islander categories because of low representation and concern that unstable subgroup estimates could be overinterpreted. We agree that this decision required clearer justification. In the revised manuscript, we now state that exclusions were made only for subgroup-specific inferential analyses where sparse cell sizes could produce unstable estimates, and we avoid drawing conclusions about groups that could not be adequately represented. We have also softened the interpretation of race/ethnicity findings and emphasized that observed differences likely reflect unequal social, occupational, environmental, and structural conditions affecting sleep opportunity rather than intrinsic group differences.

3d. *"From the results presented, it was difficult for me to validate the main conclusion, "Individuals in their 30s to 60s experience the steepest declines in both sleep duration and mood stability." There were six age groups with "30s to 60s" covering half. The plots in the figures do not help in this regard. Specifically, the independent variable is age with dependent variables sleep duration and mood stability. I cannot find any plot with age on the x-axis and the dependent variables on the y- axis. Also, I may have missed the specific statistical tests for the claim; many statistics are reported in the prose so it may be buried."*

Response: We appreciate the concern about our findings about age and the outcomes of our study. We do not have any graphics representing age as the independent variable because our study focuses on

DEPARTMENT OF PSYCHOLOGY
Life Sciences North, Room 349
1501 N. Campbell Avenue
Tucson, AZ 85724

Ofc: 520-626-2489
EMAIL: ramendoza@arizona.edu

THE UNIVERSITY OF ARIZONA
College of Science

temporal patterns and how demographics may moderate these patterns. With that in mind, we have revised our findings on these demographics to specifically refer to the patterns and not the general group average. The relevant statistics supporting these age-related patterns are reported in the Results section and our Discussion section has been prepared to be in line with our findings. As for the declines in sleep and mood, Figures 4B and 4C show the week patterns of mood shift down, then up, as age increases. The same can be seen in Figures 5C and Figures 6C and 6D, where the hourly and weekly trends of sleep duration are shifted down, then up, as age increases. Do note our figure numbers got shifted as we introduced a new Figure 1.

3e. *"Finally, I have an ethical question. It is not clear to me what ethical review was conducted. Importantly, the research is based on data provided by individuals along with their personal details. I expected at least ethical review of consent and of data governance. The authors state, "The University of Arizona Institutional Review Board determined the current study as exempt from human-subjects review." Does this mean some preliminary review was performed granting a waiver of consent and confirming re-identification risk is managed? I feel this should be clarified."*

Response: We thank the reviewer for raising this important point. We agree that the original wording was too brief and could be read as ambiguous regarding the nature of the ethical review. The University of Arizona IRB reviewed the study submission and issued a formal determination that the project did not constitute human subjects research. Specifically, the IRB notification for STUDY00001275, titled "Lumosity," states that "the committee reviewed this submission and assigned a determination of Not Human Research." Accordingly, this was not a case in which the authors independently judged the work to be exempt; rather, the institutional IRB reviewed the submission and determined that the analysis did not meet the regulatory definition of human subjects research. Because the determination was "Not Human Research," rather than "Exempt Human Subjects Research," a waiver of consent was not required in the usual sense. We have revised the Methods section to clarify this distinction.

We have also added language clarifying the data governance context. The data were obtained through Lumos Labs' Human Cognition Project data-access process, for which the investigators submitted a formal research application describing the hypotheses, requested variables, and analysis plan. The application specified that access would be limited to the subset of variables required to address the research question, including demographic variables and mood/sleep-related measures, and noted that access could only be provided to limited subsets of the Lumosity database. The dataset provided to the investigators contained anonymized/de-identified user records and did not include direct identifiers such as names, contact information, or account credentials. We have revised the manuscript to make clear that the research team analyzed a limited dataset provided by Lumos Labs, did not contact users, did not attempt re-identification, and reported only aggregate results.
