# Supplementary material for: Large-scale patterns of self-reported mood and sleep across the day and week on an online cognitive-training platform
Source: PLOS Digit Health. 2026 Sep 24;5(9):e0001754. doi: 10.1371/journal.pdig.0001754 (PMC13606931; doi:10.1371/journal.pdig.0001754)
Supplement: Supplementary file 3 [file pdig.0001754.s003.pdf]

**DEPARTMENT OF PSYCHOLOGY**
Life Sciences North, Room 349
1501 N. Campbell Avenue
Tucson, AZ 85724

Ofc: 520-626-2489
EMAIL: ramendoza@arizona.edu



THE UNIVERSITY OF ARIZONA
College of Science

August 17th, 2026

Girum Tareke Zewude, PhD
Academic Editor
*PLoS Digital Health*

**RE: Resubmission of Ms. No. PDIG-D-26-00336**

Dear Dr. Zewude:

Thank you for considering our manuscript for publication in *PLOS Digital Health* and for the opportunity to perform a second revision with regards to the comments from the reviewers. Once again, would like to thank the reviewers for their feedback regarding the significance and quality of our manuscript. Moreover, we appreciate the continued detail with the information needed to strengthen our paper. We once again assure you that we have taken these comments to heart and, in response, have rewritten parts of the manuscript with their guidance. Our point-by-point responses are presented below with the reviewer's original comments in *italics*. As presently written, we hope that you will agree that the manuscript represents a valuable contribution to the journal that will stimulate further work and discussion on the intersections between sleep and mood in large naturalistic datasets.

Sincerely,

Reina A. Mendoza, MA

DEPARTMENT OF PSYCHOLOGY
Life Sciences North, Room 349
1501 N. Campbell Avenue
Tucson, AZ 85724

Ofc: 520-626-2489
EMAIL: ramendoza@arizona.edu



THE UNIVERSITY OF ARIZONA
College of Science

## Comments to the Author

### Reviewer 1

**1a.** *The sleep duration findings have a deeper problem than the manuscript lets on. My biggest concern with this paper is about the sleep duration analyses specifically, and I want to explain why I think this is more serious than the Limitations section currently conveys. When someone logs into Lumosity at two in the afternoon and reports sleeping eight hours, they are a fundamentally different kind of person from someone logging in at seven in the morning and reporting six hours. They differ in chronotype, almost certainly in employment status, probably in lifestyle and health more broadly. The "diurnal pattern" in sleep duration that the paper describes and figures prominently in the Abstract, Results, and Discussion is therefore not really a diurnal rhythm in sleep at all. It is a diurnal rhythm in who uses the platform and when, and those different users happen to have different sleep durations. That is a selection artifact, not a sleep rhythm. The authors do acknowledge this in the Limitations, and I appreciate that. But the problem is that the rest of the paper does not honor that acknowledgment. The Abstract talks about sleep duration exhibiting "clear diurnal and weekly rhythms." The Discussion talks about "diurnal variation in sleep" and interprets afternoon reporting peaks as though they reflect something real about sleep behavior across the day. If the authors genuinely believe the time-of-report confound renders the hourly sleep findings uninterpretable as sleep rhythms, then the framing throughout needs to change consistently, not just in one paragraph at the end. If they think the findings are still meaningful despite this confound, they need to make that argument explicitly and convincingly somewhere other than a caveat buried in Limitations.*

**Response**: We thank the reviewer for raising this major concern regarding how we framed our sleep duration findings. On reflection, we agree that hourly sleep-duration findings are best interpreted as a time-of-report pattern rather than a within-person diurnal rhythm. Given our data are cross-sectional and hour of day indexes when a user logs in rather than when sleep occurs, we have revised our manuscript throughout the Title, Abstract, Introduction, Results, and Discussion, as well as relevant figure captions, to be consistent with this distinction.

Our title has been changed to "Large-scale patterns of self-reported mood and sleep across the day and week on an online cognitive-training platform" to remain neutral with respect to both mood and sleep findings, and we have replaced instances of "diurnal" or "circadian" when discussing sleep duration with "time-of-report." Our language describing mood was retained, as mood was reported at the moment of login and is therefore not subject to the same time-of-report confound as sleep duration, which asks about the prior night.

**1b.** *The design is cross-sectional and the Discussion keeps forgetting that. Each person in this dataset appears exactly once. That means the study cannot observe within-person dynamics, cannot establish*

**DEPARTMENT OF PSYCHOLOGY**
Life Sciences North, Room 349
1501 N. Campbell Avenue
Tucson, AZ 85724

Ofc: 520-626-2489
EMAIL: ramendoza@arizona.edu



THE UNIVERSITY OF ARIZONA
College of Science

*that one thing preceded another, and cannot speak to variability within a person across time. And yet the Discussion repeatedly implies all of these things. When the authors write that "shorter sleep duration was linked to greater instability in mood across the day," that is a within-person claim. When they write that "sleep may serve a restorative function through which negative affect is attenuated," that is a mechanistic claim about a process that unfolds within individuals over time. Neither of these can be demonstrated with this dataset. What can be demonstrated is that people in different sleep categories who happened to log in at different times reported different mood levels. That is genuinely interesting and worth publishing, but it is a different and more modest claim than what the Discussion is currently making. I would ask the authors to go through the Discussion carefully and revise any language that implies directionality, mechanism, or within-person change.*

**Response:** We thank the reviewer for this important point and agree the Discussion does not consistently respect the limits of a cross-sectional design. On re-reading, we identified several passages where our language implies within-person dynamics or causal mechanisms that a single-observation-per-user dataset cannot support. We have revised these throughout and ensured our claims describe cross-sectional associations between user data, rather than implying within-person change, causal relationships, or any psychological mechanisms that we cannot establish here. Examples include:

The claim that "shorter sleep duration was linked to greater hour-to-hour variability in reported mood" described variability in hourly *group averages* across users, not within any one person's day. We have reworded this and similar passages to make clear the variability described is across users sampled at different hours, not within-person fluctuations.

The claim that women's shorter morning sleep "may contribute to less favorable mood upon waking" was revised to describe a pattern of cross-sectional association between reported sleep and reported mood, rather than a consequence of waking.

The passage proposing "sleep may serve a restorative function that attenuates negative affect" both describe mechanisms that reference other studies and theories, but we have tightened the writing so that it is clear we are referring to outside literature's proposed mechanisms and not testing them directly in our data.

**1c.** *The paper essentially never discusses practical effect sizes. With nearly half a million observations, the study has more statistical power than almost any analysis would ever need. Under these conditions, p-values are essentially meaningless as indicators of whether a finding matters. Every effect, no matter how small, will clear any conventional significance threshold. The manuscript presents table after table of F-statistics and $p < .0001$ results without consistently reporting effect sizes that would let a reader judge whether any of this actually matters in practice. The β coefficients are helpful in some places, but they are unstandardized and reported on bounded scales, which makes*

**DEPARTMENT OF PSYCHOLOGY**
Life Sciences North, Room 349
1501 N. Campbell Avenue
Tucson, AZ 85724

Ofc: 520-626-2489
EMAIL: ramendoza@arizona.edu



THE UNIVERSITY OF ARIZONA
College of Science

*them hard to interpret without more context. The authors should report standardized effect sizes for the primary findings and devote some attention to the Discussion to which effects are large enough to be practically meaningful and which, despite their overwhelming statistical significance, may be quite small.*

**Response:** We thank the reviewer for this important point and have made substantial additions throughout the manuscript to address it.

First, our Methods section now has a paragraph describing our approach to reporting effect sizes. We computed omega-squared and partial omega-squared for each main effect and interaction term. Effect sizes were computed from the unadjusted (Model 1) version of the test, consistent with our existing approach to coefficient extraction. We also noted that conventional benchmarks for effect size were developed in contexts distinct from large-scale, naturalistic behavioral data of the kind analyzed here, and as such we interpret their magnitude accordingly.

Second, our Results section now has the relevant effect sizes along every set of statistics reported, in Tables 1 and 2 next to the Model 1 p-values, and we also directly stated any main-effect effect sizes for our predictor variables, making sure to label them explicitly to avoid ambiguity (omnibus main effect)

Finally, our Discussion section now has a paragraph immediately following its introduction that directly addresses the limited informativeness of our p-values with a sample size this large. We also state that most temporal and demographic effects are pretty small by conventional standard, many so low to the point they appear almost non-existent, all while the association between mood and sleep duration is comparatively large ($\omega^2_p \approx .04–.05$) and represents our most practically substantial finding. We also mentioned this framework within some of our paragraphs describing mood and sleep relationships as well as our paragraph on the disparities in race / ethnicity for sleep duration, where although the effect size was small ($\omega^2_p \approx .004$), is still accompanied by a practically meaningful group difference of approximately 20 minutes of sleep report between Black and White users.

**1d.** *The sample is more limited than the public health framing suggests. The Limitations section acknowledges that the sample skews White, female, and highly educated. But the Abstract, the Introduction, and especially the Conclusion speak in broad terms about "public health significance," "population-level windows of vulnerability," and "sleep inequity" without adequately flagging how much these conclusions depend on a self-selected group of cognitive-training app users from over a decade ago. This matters especially for the racial and ethnic disparity findings. Black users who were using Lumosity in 2013 were almost certainly not representative of Black Americans broadly. They were likely skewed toward higher education, greater digital access, and stronger health motivation, all of which would tend to attenuate observed sleep disparities relative to the true population-level gap. The paper does not discuss this possibility at all, and it should.*

DEPARTMENT OF PSYCHOLOGY
Life Sciences North, Room 349
1501 N. Campbell Avenue
Tucson, AZ 85724

Ofc: 520-626-2489
EMAIL: ramendoza@arizona.edu



THE UNIVERSITY OF ARIZONA
College of Science

**Response:** We thank the reviewer for this important point. We have revised the Abstract, Introduction, Discussion, Limitations, and Conclusion sections to more clearly flag the sample's limitations, specifically for the race / ethnicity findings and the self-selection within a cognitive-training platform. We noted clearly how these limitations may bias our estimates to understate true population-level disparities. For instance:

- Our revised Abstract has toned down claims about "public health significance" and generalizability in the population-level.
- Our revised Introduction notes on sections about "population-level windows of vulnerability" that the sample is a self-selected group of app users
- Our revised Discussion and Limitations bring up the disparity in samples sizes for race / ethnicity
- Our revised Conclusion's language on "sleep inequity" or "public health significance" is reframed as hypothesis-generating rather than population-level

**1e.** *The data are from 2013 to 2014 and that deserves more than a passing mention. The authors note the data collection period in the Methods and leave it at that. But these data are more than ten years old. Smartphone penetration, sleep patterns, the demographics of who uses health and wellness apps, and the social context surrounding sleep and mental health have all changed substantially since then. Several of the papers cited in the Discussion are from 2020, 2021, 2022, and beyond. Asking whether findings from a 2013 early-adopter Lumosity population generalize to contemporary populations referenced in recent literature is a legitimate question that deserves at least a paragraph of honest discussion.*

**Response:** We agree with the reviewer comments here just like with our sample demographic point on 1d and have made changes addressing this time gap within our Limitations and Discussion sections:

First, our revised Discussion has a sentence in the race / ethnicity disparities paragraph noting our comparisons to recently published literature span more than a decade relative to our data collection, with a pointer to the Limitations for more nuance about this.

Second, our revised Limitations has a new paragraph placed after the race / ethnicity paragraph acknowledging the data's age and the broad changes in sleep and mental health ever since (including pandemic-related shifts). We distinguished between the findings which we believed to be more robust to this gap (such as the hourly and weekly patterns of mood and sleep) and those which may be more vulnerable to it (such as the demographic disparities). We call for replication using more recently collected data to further strengthen the present findings.

**DEPARTMENT OF PSYCHOLOGY**
Life Sciences North, Room 349
1501 N. Campbell Avenue
Tucson, AZ 85724

Ofc: 520-626-2489
EMAIL: ramendoza@arizona.edu



THE UNIVERSITY OF ARIZONA
College of Science

Finally, our revised Conclusion has a closing sentence noting the age of the data and how future work is important to confirm to what extent this data retains relevance in contemporary populations.

**1f.** *The suicide prevention implication is stretched further than the data can reach. I recognize that some of the authors have published important work on nocturnal wakefulness and suicide risk, and I think the connection they are drawing is scientifically plausible. But the people logging into a brain-training app at three in the morning and reporting low mood are not a clinically representative sample of individuals at suicide risk. Using their mood ratings to support arguments about overnight crisis service capacity is an inferential leap that the paper does not flag clearly enough as speculative. The implication is not wrong, but it is presented with more confidence than the evidence here actually warrants.*

**Response**: We agree with this assessment and have revised the Abstract, Discussion, and Conclusion to clearly distinguish between our finding (a robust pattern of nocturnal negative affect in a large, nonclinical sample) and the clinical / policy implications for suicide risk and crisis-service capacity, which our data cannot directly establish.

First our Abstract now specifies that our sample is nonclinical and cannot directly speak to clinical risk, replacing the language that presents the suicide-prevention implication without qualification.

Second, our Discussion now contains a caveat section directly after the discussion of overnight negative affect and suicide-risk literature, where we clarified Lumosity users logging in overnight are nonclinical, self-selected, and not assessed for elevated suicide risk. We note our findings are suggestive of the underlying risks shown in the literature rather than confirmation and that low mood in our users reporting in the middle of the night does not establish the experience of clinically significant risk.

Finally, our Conclusion is revised so the paragraph on nighttime negative affect and suicide risk to state that our sample's mood ratings cannot be used to directly estimate suicide risk or crisis-service needs. Once again, we emphasized the data as a way to show support for a pattern established in clinical and epidemiological research, but not as direct evidence of policy or interventions.

**1g.** *The Age by Weekday interaction did not survive full model adjustment. Table 1 shows clearly that this interaction was significant in Models 1 and 2 but disappeared entirely in Model 3. The authors mention this in the Results, which is appropriate. But the Discussion continues to treat age-related mood patterns across the week as a substantive finding without acknowledging that the effect was not robust to adjustment for sleep duration. If an interaction cannot survive covariate adjustment in a half-million person sample, that is telling you something important about its fragility, and the Discussion should reflect that honestly.*

DEPARTMENT OF PSYCHOLOGY
Life Sciences North, Room 349
1501 N. Campbell Avenue
Tucson, AZ 85724

Ofc: 520-626-2489
EMAIL: ramendoza@arizona.edu



THE UNIVERSITY OF ARIZONA
College of Science

**Response:** We agree with the reviewer on this being an oversight. While the Results section correctly reports that the age by weekday interaction was not significant in the fully adjusted model, the Discussion did not clearly distinguish this from the main effect of age on mood, which remained significant throughout. We have revised the Discussion to make this distinction explicit. In the paragraph discussing age-related mood patterns, we added a sentence clarifying that the observed U-shaped pattern reflects a main effect of age on mood, not a moderating effect of age on weekly mood rhythms. We note that although the age by weekday interaction was significant in the unadjusted and partially adjusted models, it did not remain significant after adjustment for sleep duration in the fully specified model, and that given the size of the present sample, this indicates the interaction was not robust. We now interpret age as shaping overall mood level rather than the shape of weekly mood variation, consistent with the reviewer's point that a null result in a sample of this size is itself informative.

**1h.** *Several figure captions interpret rather than describe. Captions are supposed to tell readers what they are looking at, not explain why it occurs. Describing the mood dip among nine-hour sleepers as reflecting "the potential drawbacks of oversleeping on affect" is a causal interpretation. Attributing stable weekend sleep among older adults to "relief from work-duties and retirement" is a speculation, given that employment status was never measured. These kinds of interpretations belong in the Discussion, conditionally and with appropriate hedging, not in figure captions stated as though they are established.*

**Response:** We agree that the captions should describe what is plotted rather than explain why the pattern exists and thank the reviewer for identifying some clear instances of this. On review, we found the same problem in several other captions beyond the ones the reviewer flagged, so we have revised all figure captions throughout to remove interpretative and causal language, moving any genuine interpretation to the Discussion section where it can be properly cited and framed. Here's an example of how we changed the caption for Figure 3B for mood and sleep duration across hour bins:

<u>Old Caption</u>: Hourly mood ratings averaged across days, plotted for each hour bin and separated by sleep duration category. Users reporting longer sleep durations reported higher overall mood, with 8 hours at the highest. Users reporting 5 hours of sleep displayed a lack of rhythm with sporadic peaks and troughs throughout the day, and this variability stabilized as sleep duration increased. Interestingly, users reporting 9 hours of sleep showed a decrease in mood reports compared to 8 hours of sleep, which reflects the potential drawbacks of oversleeping on affect.

<u>New Caption</u>: Hourly mood ratings averaged across days, plotted for each hour bin and separated by sleep duration category. Users reporting longer sleep duration reported higher overall mood, **with the 8-hour group highest.** Among users reporting 5 hours of sleep, **hourly mood averages varied more**

**DEPARTMENT OF PSYCHOLOGY**
Life Sciences North, Room 349
1501 N. Campbell Avenue
Tucson, AZ 85724

Ofc: 520-626-2489
EMAIL: ramendoza@arizona.edu



THE UNIVERSITY OF ARIZONA
College of Science

**sharply by report time**; this variation was smaller among users reporting longer sleep durations. Users reporting 9 hours of sleep showed lower mood on average than the 8-hour group.

**1i.** *The Model 3 covariate choice raises a subtle but real concern. Including mood as a categorical predictor in the sleep duration models, and sleep as a categorical predictor in the mood models, is an interesting and defensible choice as a robustness check. But the justification offered is not quite complete. If mood and sleep are genuinely bidirectionally related, as the authors argue throughout the paper, then including one as a covariate when modeling the other can introduce overcontrol or collider bias depending on the assumed causal structure. The authors should either articulate the causal logic behind Model 3 more explicitly or clarify that it is purely a descriptive robustness check rather than a causally motivated adjustment.*

**Response:** We thank the reviewer for raising this point, which we agree needed clarification. Model 3 was included as a robustness check in response to a request from an earlier round of review, and we did not conduct a formal mediation or moderation analysis to establish the direction or mechanism of the relationship between mood and sleep duration. We have revised the Analysis section and the notes accompanying Table 1 and Table 2 to state this explicitly.
- The new Analysis has a paragraph clarifying that, because mood and sleep duration are plausibly bidirectionally related, including one as a covariate when modeling the other does not represent a casually motivated adjustment. We state explicitly that no formal mediation or moderation analysis was performed, and that Model 3 was included as a descriptive robustness check to assess whether associations of interest persisted after accounting for the complementary behavioral outcome. We clarify now that Model 3 coefficients should be interpreted as evidence of robustness to this adjustment, not as casually identified effects.
- The New Table 1 and Table 2 notes now have a sentence to both stating Model 3 serves as a descriptive robustness check and is not intended to represent an adjusted estimate, so this clarification is visible to readers to reference the tables directly.

**1j.** *The Monday blues interpretation is partly a product of the comparison structure. The paper presents low Monday mood as a substantive finding and connects it to established "Monday blues" literature, which is reasonable. But it is worth noting that in a weighted effect coding framework where weekend mood is high, adjacent weekdays will appear relatively low partly by mathematical construction. The finding is real, but the framing slightly overstates how independent it is from the coding approach.*

**Response:** We agree with this point and have revised both the Discussion text and the Fig 2 caption to address it. In an earlier version of the manuscript, we had referenced the "Monday blues" literature directly in a figure caption; this framing has since been removed, but we recognize that the underlying

**DEPARTMENT OF PSYCHOLOGY**
Life Sciences North, Room 349
1501 N. Campbell Avenue
Tucson, AZ 85724

Ofc: 520-626-2489
EMAIL: ramendoza@arizona.edu



THE UNIVERSITY OF ARIZONA
College of Science

interpretive issue the reviewer raises extends beyond that specific phrase to how we describe weekday mood patterns more generally.

Our Discussion now has text clarifying that, because weighted effect coding expresses each day's coefficient relative to the weighted average across the full week, the appearance of lower mood on weekdays, particularly early in the week, is partly a function of this comparison structure rather than necessarily reflecting a day-specific effect. We now note that the overall weekly pattern (elevated weekend mood, lower weekday mood) remains a robust and substantive finding, but that the relative magnitude of any single weekday's coefficient should be interpreted with this coding structure in mind.

Our Fig 2 caption has been revised to describe the pattern as occurring “early in the work week” rather than singling out “Monday mornings” specifically, such that the caption does not inadvertently imply a day-specific effect, all while our Discussion text now explicitly cautions against it.
