# Supplementary material for: Large-scale patterns of self-reported mood and sleep across the day and week on an online cognitive-training platform
Source: PLOS Digit Health. 2026 Sep 24;5(9):e0001754. doi: 10.1371/journal.pdig.0001754 (PMC13606931; doi:10.1371/journal.pdig.0001754)
Supplement: Peer review history

# Peer review: Decision Letter 0

**DOI:** 10.1371/journal.pdig.0001754.r001

**Authors:** Leo Anthony Celi, Girum Tareke Zewude

27 Apr 2026

Response to Reviewers Revised Manuscript with Track Changes Manuscript **Journal Requirements:**

1. Please send a completed 'Competing Interests' statement, including any COIs declared by your co-authors. If you have no competing interests to declare, please state "The authors have declared that no competing interests exist". Otherwise please declare all competing interests beginning with the statement "I have read the journal's policy and the authors of this manuscript have the following competing interests:"

2. For studies involving third-party data, we encourage authors to share any data specific to their analyses that they can legally distribute. PLOS recognizes, however, that authors may be using third-party data they do not have the rights to share. When third-party data cannot be publicly shared, authors must provide all information necessary for interested researchers to apply to gain access to the data. (https://journals.plos.org/plosone/s/data-availability#loc-acceptable-data-access-restrictions)

For any third-party data that the authors cannot legally distribute, they should include the following information in their Data Availability Statement upon submission:

1) A description of the data set and the third-party source

2) If applicable, verification of permission to use the data set

3) Confirmation of whether the authors received any special privileges in accessing the data that other researchers would not have

4) All necessary contact information others would need to apply to gain access to the data

3. We ask that a manuscript source file is provided at Revision. Please upload your manuscript file as a .doc, .docx, .rtf or .tex.

**Additional Editor Comments (if provided):**

Thank you very much for the opportunity to review your manuscript for PLOS Digital Health.

I have carefully read your manuscript and the reviewers’ comments. The reviewers provided detailed and rigorous feedback, particularly in the reports from Reviewer 1 and Reviewer 3. I have shared the reviewers’ comments and recommendations with the editorial office.

I appreciate your efforts and the work you have put into this submission.

Sincerely,

**Reviewers' Comments:**

**Comments to the Author**

1. Does this manuscript meet PLOS Digital Health’s publication criteria?

Reviewer #1: Partly

Reviewer #2: Yes

Reviewer #3: Partly

**********

2. Has the statistical analysis been performed appropriately and rigorously?

Reviewer #1: No

Reviewer #2: N/A

Reviewer #3: I don't know

**********

3. Have the authors made all data underlying the findings in their manuscript fully available (please refer to the Data Availability Statement at the start of the manuscript PDF file)?

The PLOS Data policy

Reviewer #1: No

Reviewer #2: Yes

Reviewer #3: No

**********

4. Is the manuscript presented in an intelligible fashion and written in standard English?

Reviewer #1: Yes

Reviewer #2: Yes

Reviewer #3: Yes

**********

**Reviewer #1:**  The manuscript presents a large-scale analysis of self-reported mood and sleep data, which is valuable. However, the description of participant inclusion and exclusion criteria requires further clarification. The rationale for excluding certain age groups (15–19 and 80–90) and specific ethnic categories such as Native Americans and Pacific Islanders is not fully justified. The authors should explain whether these exclusions were based on sample size limitations or potential biases, and discuss the implications for generalizability of the findings. Without this clarification, it is difficult to assess how representative the sample is of the broader population.

The measurement of mood and sleep duration, while described, lacks sufficient information on validation. The five-point mood scale and the binned sleep duration categories may not fully capture the variability in affective states or sleep patterns. The authors should provide evidence that these self-report measures are reliable and, if possible, validated against objective data such as actigraphy or clinical assessments. This would strengthen confidence in the accuracy of the outcomes analyzed.

The manuscript mentions rounding the extreme sleep categories (≤5 hours and 9+ hours) to 5 and 9, but does not discuss how this transformation might influence statistical analyses. The authors should elaborate on potential effects on variance, regression coefficients, and interpretation of extreme sleep durations, particularly since these extremes may carry important behavioral or clinical implications.

The description of regression models (Models 1–3) is somewhat confusing and dense. While the distinction between unadjusted, adjusted, and fully specified models is noted, a clearer presentation is needed. Including a summary table or diagram that shows the main effect, covariates, complementary outcome, and interaction terms for each model would enhance readability and allow readers to follow the statistical strategy more easily.

Weighted effect coding is applied for hour and weekday variables, but the rationale for choosing this method over standard dummy coding is not fully explained. The authors should justify why weighted effect coding was selected and discuss how it influences the interpretation of regression coefficients compared to unweighted or reference-based coding methods. This will help clarify the analytical decisions for the reader.

The modeling of interactions, especially between hour/weekday and demographic or behavioral factors, is central to the analysis. However, the criteria for including interaction terms in Models 2 and 3 are not clearly described. The authors should specify whether only interactions significant in Model 1 were carried forward, and whether any adjustments for multiple comparisons were applied. Clearer documentation of the interaction modeling strategy would improve reproducibility and interpretability.

The use of one-way and two-way ANOVAs to assess main effects and interactions requires additional justification. Given that mood and sleep measures are ordinal or interval variables, the authors should clarify why ANOVA is appropriate and whether assumptions such as normality and homogeneity of variance were tested. Addressing these points will strengthen the methodological rigor of the statistical analyses.

The temporal aggregation of data into hourly and weekly bins is not fully described. It is unclear how multiple entries from the same user or uneven login times were handled. The authors should clarify whether data were aggregated per user or pooled across users, and whether weighting was applied to account for unequal contributions. This information is critical for understanding the derivation of diurnal and weekly patterns.

Demographic covariates, including age, gender, and ethnicity, are treated categorically. The authors should provide a rationale for using categorical bins rather than continuous measures, particularly for age. Additionally, potential collinearity among covariates should be assessed and reported, as it could influence the interpretation of regression coefficients and interactions.

Finally, while the manuscript notes that analyses were performed in R and Python, it does not provide sufficient detail for reproducibility. The authors should specify packages, functions, and versions, and ideally provide a reproducible workflow or code repository. This transparency is essential given the complexity of the modeling approach and the large dataset analyzed.

**Reviewer #2:**  Clinically relevant paper of large-scale diurnal and weekly patterns of self-reported mood and sleep on an online cognitive-training platform. Clinically relevant paper of large-scale diurnal and weekly patterns of self-reported mood and sleep on an online cognitive-training platform.

**Reviewer #3:**  The paper reports on analysis of data collected by a brain-training app running on smart devices. The authors analysed data from nearly 0.5 million individuals, examining self-reported mood and sleep duration, factored by aged, time-of-day, and other variables. The primary research question is to resolve previously contradictory findings about sleep architecture and aging. Secondary questions regard diurnal and weekly variations in mood and sleep duration. They report three conclusions. (1) Individuals in their 30s to 60s experience the steepest declines in both sleep duration and mood stability. (2) Mood follows a diurnal pattern. (3) They confirm that Black and Hispanic people report shorter sleep durations, compared to White people.

Unfortunately, I could not get a strong sense of importance of the research questions or findings from the paper. The authors tie their findings to suicide prevention, workplace policies, and socioeconomic disadvantage. These are emotionally powerful and the authors cite relevant works [32-36, 59, 60]. However, the paper could provide more analysis of the literature and deeper explanation to make the importance of the work more clear. The authors also motivate the research citing contradictory findings about sleep architecture and aging. However, that literature is not critically examined.

I have concerns about the methodology. I could not find some important characteristics of the dataset, such as: (a) what time period the data covers, (b) what were the triggers prompting users to self-report, (c) the numbers of mood and sleep reports, and (d) basic demographic statistics of the dataset. My main methodological concern is that simple things like record numbers or data collection biases may contribute to some findings. The nature of the trigger (b) may introduce significant biases into the analysis. Additionally, for sleep duration data I don't have a clear appreciation of why time-of-day has a clean meaning. The authors already draw attention to biases from users self-selecting to be in the study. If data collection was self-triggered this introduces a potentially devastating bias - one that is not measured and not discussed. Additionally, it is not clear to me what effects on findings there may be from shift work and unconventional sleeping habits.

I am also a little confused about data on Black, Hispanic, White, etc. Firstly, it is not clear to me what these categories are. E.g., are they race or ethnicity, or some conflation of the two. Also, I could not see the scientific need for considering this variable; there was no associated research question and the only relevant conclusion merely confirmed existing knowledge. Given the questionable value of this variable, it may be better to simply ignore it for analysis. This also eliminates any need to discard categories Native American and Pacific Islanders due to underrepresentation.

From the results presented, it was difficult for me to validate the main conclusion, "Individuals in their 30s to 60s experience the steepest declines in both sleep duration and mood stability." There were six age groups with "30s to 60s" covering half. The plots in the figures do not help in this regard. Specifically, the independent variable is age with dependant variables sleep duration and mood stability. I cannot find any plot with age on the x-axis and the dependant variables on the y-axis. Also, I may have missed the specific statistical tests for the claim; many statistics are reported in the prose so it may be buried.

Finally, I have an ethical question. It is not clear to me what ethical review was conducted. Importantly, the research is based on data provided by individuals along with their personal details. I expected at least ethical review of consent and of data governance. The authors state, "The University of Arizona Institutional Review Board determined the current study as exempt from human-subjects review." Does this mean some preliminary review was performed granting a waiver of consent and confirming re-identification risk is managed? I feel this should be clarified.

To be acceptable, I recommend the following.

1) Consider strengthening the motivation for the research questions, e.g., by discussing the literature with respect to suicide prevention, workplace policies, and socioeconomic disadvantage. Similarly, consider more precisely discussing the missing knowledge that the research satisfies, e.g., a conflicting understanding of age-related sleep habits.

2) Make clear the circumstances triggering users to produce a self-report. Discuss the possible impact of this on the findings.

3) Make clear the time period of the data collection.

4) Include histograms of record counts per individual, perhaps grouped by key variables such as time-of-day and age.

5) Explain why time-of-day is a meaningful variable for self-reports, especially for sleep duration. Discuss possible effects on findings where reporting time differs from sleep time. Consider also discussing the relevance of shift work and other non-standard sleep habits.

6) Include histograms of demographic variables and other basic variables.

7) Consider removing analysis based on race and ethnicity. Otherwise, clarify the definition of such variables and properly motivate and state the relevant research questions.

8) Consider making the results that feed into the main conclusion more clear. E.g., include plots that directly relate to the conclusions by having independent variables on the x-axis and the dependant variables on the y-axis. Consider making the overall reporting of statistics more concise with emphasis on relevance to the conclusions.

9) Clarify the ethical review of the research, particularly regarding any waiver of consent, data governance, and reidentification risks.

**********

what does this mean?). If published, this will include your full peer review and any attached files.

**Do you want your identity to be public for this peer review?**  If you choose “no”, your identity will remain anonymous but your review may still be made public.

For information about this choice, including consent withdrawal, please see our Privacy Policy

Reviewer #1: **Yes:**  Md. Abu Bokkor Shiddik

Reviewer #2: No

Reviewer #3: No

**********

**Figure resubmission:**

**Reproducibility:**

To enhance the reproducibility of your results, we recommend that authors of applicable studies deposit laboratory protocols in protocols.io, where a protocol can be assigned its own identifier (DOI) such that it can be cited independently in the future. Additionally, PLOS ONE offers an option to publish peer-reviewed clinical study protocols. Read more information on sharing protocols at https://plos.org/protocols?utm_medium=editorial-email&utm_source=authorletters&utm_campaign=protocols

# Author comment: Author response to Decision Letter 1

**DOI:** 10.1371/journal.pdig.0001754.r002

25 May 2026

Attachment Submitted filename: Response_to_Reviewers.pdf

# Peer review: Decision Letter 1

**DOI:** 10.1371/journal.pdig.0001754.r003

**Authors:** Leo Anthony Celi, Girum Tareke Zewude

19 Jul 2026

Response to ReviewersRevised Manuscript with Track ChangesManuscript**Journal Requirements:** **Additional Editor Comments (if provided):** **Reviewers' Comments:**

**Comments to the Author**

Reviewer #1: (No Response)

Reviewer #2: All comments have been addressed

**********

publication criteria?

Reviewer #1: No

Reviewer #2: Yes

**********

3. Has the statistical analysis been performed appropriately and rigorously?

Reviewer #1: No

Reviewer #2: Yes

**********

4. Have the authors made all data underlying the findings in their manuscript fully available (please refer to the Data Availability Statement at the start of the manuscript PDF file)?

The PLOS Data policy

Reviewer #1: No

Reviewer #2: Yes

**********

5. Is the manuscript presented in an intelligible fashion and written in standard English?

Reviewer #1: Yes

Reviewer #2: (No Response)

**********

Reviewer #1: General Assessment

I want to start by acknowledging that this is a genuinely interesting dataset and the research questions being asked are worthwhile. Nearly half a million observations of directly self-reported mood and sleep is not something you come across often, and the authors have clearly put serious work into both the analysis and the revision. The response to prior reviewers was thorough and the manuscript is meaningfully improved. That said, I have several concerns that I think need to be addressed before this paper is ready for publication, and some of them are fairly fundamental to how the findings are being framed.

Major Concerns

The sleep duration findings have a deeper problem than the manuscript lets on

My biggest concern with this paper is about the sleep duration analyses specifically, and I want to explain why I think this is more serious than the Limitations section currently conveys.

When someone logs into Lumosity at two in the afternoon and reports sleeping eight hours, they are a fundamentally different kind of person from someone logging in at seven in the morning and reporting six hours. They differ in chronotype, almost certainly in employment status, probably in lifestyle and health more broadly. The "diurnal pattern" in sleep duration that the paper describes and figures prominently in the Abstract, Results, and Discussion is therefore not really a diurnal rhythm in sleep at all. It is a diurnal rhythm in who uses the platform and when, and those different users happen to have different sleep durations. That is a selection artifact, not a sleep rhythm.

The authors do acknowledge this in the Limitations, and I appreciate that. But the problem is that the rest of the paper does not honor that acknowledgment. The Abstract talks about sleep duration exhibiting "clear diurnal and weekly rhythms." The Discussion talks about "diurnal variation in sleep" and interprets afternoon reporting peaks as though they reflect something real about sleep behavior across the day. If the authors genuinely believe the time-of-report confound renders the hourly sleep findings uninterpretable as sleep rhythms, then the framing throughout needs to change consistently, not just in one paragraph at the end. If they think the findings are still meaningful despite this confound, they need to make that argument explicitly and convincingly somewhere other than a caveat buried in Limitations.

The design is cross-sectional and the Discussion keeps forgetting that

Each person in this dataset appears exactly once. That means the study cannot observe within-person dynamics, cannot establish that one thing preceded another, and cannot speak to variability within a person across time. And yet the Discussion repeatedly implies all of these things.

When the authors write that "shorter sleep duration was linked to greater instability in mood across the day," that is a within-person claim. When they write that "sleep may serve a restorative function through which negative affect is attenuated," that is a mechanistic claim about a process that unfolds within individuals over time. Neither of these can be demonstrated with this dataset. What can be demonstrated is that people in different sleep categories who happened to log in at different times reported different mood levels. That is genuinely interesting and worth publishing, but it is a different and more modest claim than what the Discussion is currently making. I would ask the authors to go through the Discussion carefully and revise any language that implies directionality, mechanism, or within-person change.

The paper essentially never discusses practical effect sizes

With nearly half a million observations, the study has more statistical power than almost any analysis would ever need. Under these conditions, p-values are essentially meaningless as indicators of whether a finding matters. Every effect, no matter how small, will clear any conventional significance threshold. The manuscript presents table after table of F-statistics and p < .0001 results without consistently reporting effect sizes that would let a reader judge whether any of this actually matters in practice. The β coefficients are helpful in some places, but they are unstandardized and reported on bounded scales, which makes them hard to interpret without more context. The authors should report standardized effect sizes for the primary findings and devote some attention in the Discussion to which effects are large enough to be practically meaningful and which, despite their overwhelming statistical significance, may be quite small.

The sample is more limited than the public health framing suggests

The Limitations section acknowledges that the sample skews White, female, and highly educated. But the Abstract, the Introduction, and especially the Conclusion speak in broad terms about "public health significance," "population-level windows of vulnerability," and "sleep inequity" without adequately flagging how much these conclusions depend on a self-selected group of cognitive-training app users from over a decade ago. This matters especially for the racial and ethnic disparity findings. Black users who were using Lumosity in 2013 were almost certainly not representative of Black Americans broadly. They were likely skewed toward higher education, greater digital access, and stronger health motivation, all of which would tend to attenuate observed sleep disparities relative to the true population-level gap. The paper does not discuss this possibility at all, and it should.

The data are from 2013 to 2014 and that deserves more than a passing mention

The authors note the data collection period in the Methods and leave it at that. But these data are more than ten years old. Smartphone penetration, sleep patterns, the demographics of who uses health and wellness apps, and the social context surrounding sleep and mental health have all changed substantially since then. Several of the papers cited in the Discussion are from 2020, 2021, 2022, and beyond. Asking whether findings from a 2013 early-adopter Lumosity population generalize to contemporary populations referenced in recent literature is a legitimate question that deserves at least a paragraph of honest discussion.

Moderate Concerns

The suicide prevention implication is stretched further than the data can reach

I recognize that some of the authors have published important work on nocturnal wakefulness and suicide risk, and I think the connection they are drawing is scientifically plausible. But the people logging into a brain-training app at three in the morning and reporting low mood are not a clinically representative sample of individuals at suicide risk. Using their mood ratings to support arguments about overnight crisis service capacity is an inferential leap that the paper does not flag clearly enough as speculative. The implication is not wrong, but it is presented with more confidence than the evidence here actually warrants.

The Age by Weekday interaction did not survive full model adjustment

Table 1 shows clearly that this interaction was significant in Models 1 and 2 but disappeared entirely in Model 3. The authors mention this in the Results, which is appropriate. But the Discussion continues to treat age-related mood patterns across the week as a substantive finding without acknowledging that the effect was not robust to adjustment for sleep duration. If an interaction cannot survive covariate adjustment in a half-million person sample, that is telling you something important about its fragility, and the Discussion should reflect that honestly.

Several figure captions interpret rather than describe

Captions are supposed to tell readers what they are looking at, not explain why it occurs. Describing the mood dip among nine-hour sleepers as reflecting "the potential drawbacks of oversleeping on affect" is a causal interpretation. Attributing stable weekend sleep among older adults to "relief from work-duties and retirement" is a speculation, given that employment status was never measured. These kinds of interpretations belong in the Discussion, conditionally and with appropriate hedging, not in figure captions stated as though they are established.

Minor Concerns

The Model 3 covariate choice raises a subtle but real concern

Including mood as a categorical predictor in the sleep duration models, and sleep as a categorical predictor in the mood models, is an interesting and defensible choice as a robustness check. But the justification offered is not quite complete. If mood and sleep are genuinely bidirectionally related, as the authors argue throughout the paper, then including one as a covariate when modeling the other can introduce overcontrol or collider bias depending on the assumed causal structure. The authors should either articulate the causal logic behind Model 3 more explicitly or clarify that it is purely a descriptive robustness check rather than a causally motivated adjustment.

The Monday blues interpretation is partly a product of the comparison structure

The paper presents low Monday mood as a substantive finding and connects it to established "Monday blues" literature, which is reasonable. But it is worth noting that in a weighted effect coding framework where weekend mood is high, adjacent weekdays will appear relatively low partly by mathematical construction. The finding is real, but the framing slightly overstates how independent it is from the coding approach.

In summary

I want to be clear that I think this paper is worth publishing. The dataset is rare, the questions are important, and the demographic breadth of the findings is a genuine contribution to the literature on sleep and mood in naturalistic settings. My concerns are not about whether the work should eventually appear in print. They are about whether the paper as currently written is honest about what this particular design can and cannot show. The sleep duration and diurnal framing needs to be rethought from the Abstract onward, not just footnoted in Limitations. The Discussion needs to be brought into alignment with what a single-observation cross-sectional design actually supports. And the effect size question needs to be taken seriously rather than implicitly delegated to statistical significance. I hope the authors will engage with these points in revision because I think doing so will make this a stronger and more durable contribution to the field.

Reviewer #2: Clinically relevant paper on large-scale diurnal and weekly patterns of self-reported mood and sleep on an online Cognitive-Training Platform.

Clinically relevant paper on large-scale diurnal and weekly patterns of self-reported mood and sleep on an online Cognitive-Training Platform.

**********

what does this mean?). If published, this will include your full peer review and any attached files.

**Do you want your identity to be public for this peer review?**  If you choose “no”, your identity will remain anonymous but your review may still be made public.

For information about this choice, including consent withdrawal, please see our Privacy Policy

Reviewer #1: **Yes:**  Md. Abu Bokkor Shiddik

Reviewer #2: No

**********

**Figure resubmission:**

**Reproducibility:**

To enhance the reproducibility of your results, we recommend that authors of applicable studies deposit laboratory protocols in protocols.io, where a protocol can be assigned its own identifier (DOI) such that it can be cited independently in the future. Additionally, PLOS ONE offers an option to publish peer-reviewed clinical study protocols. Read more information on sharing protocols at https://plos.org/protocols?utm_medium=editorial-email&utm_source=authorletters&utm_campaign=protocols

# Author comment: Author response to Decision Letter 2

**DOI:** 10.1371/journal.pdig.0001754.r004

18 Aug 2026

Attachment Submitted filename: Response to Reviewers.pdf

# Editor report: Decision Letter 2

**DOI:** 10.1371/journal.pdig.0001754.r005

**Authors:** Girum Zewude

6 Sep 2026

Large-scale patterns of self-reported mood and sleep across the day and week on an online cognitive-training platform

PDIG-D-26-00336R2

Dear Mendoza,

We are pleased to inform you that your manuscript 'Large-scale patterns of self-reported mood and sleep across the day and week on an online cognitive-training platform' has been provisionally accepted for publication in PLOS Digital Health.

Before your manuscript can be formally accepted you will need to complete some formatting changes, which you will receive in a follow-up email from a member of our team.

Please note that your manuscript will not be scheduled for publication until you have made the required changes, so a swift response is appreciated.

IMPORTANT: The editorial review process is now complete. PLOS will only permit corrections to spelling, formatting or significant scientific errors from this point onwards. Requests for major changes, or any which affect the scientific understanding of your work, will cause delays to the publication date of your manuscript.

If your institution or institutions have a press office, please notify them about your upcoming paper to help maximize its impact. If they'll be preparing press materials, please inform our press team as soon as possible -- no later than 48 hours after receiving the formal acceptance. Your manuscript will remain under strict press embargo until 2 pm Eastern Time on the date of publication. For more information, please contact digitalhealth@plos.org.

Thank you again for supporting Open Access publishing; we are looking forward to publishing your work in PLOS Digital Health.

Best regards,

Girum Tareke Zewude, PhD

Section Editor

PLOS Digital Health

***********************************************************

**Additional Editor Comments (if provided):**

Dear Authors,

I would like to appreciate and thank you for your careful revision and scientific response. I would also like to add one comment and kindly request that you carefully follow the PLOS Digital Health Journal guidelines in the final submission.

My congratulations on the acceptance of your manuscript. It addresses the reviewers’ comments in an interesting and valuable way.

**Reviewer Comments (if any, and for reference):**
